# Pathogenesis and transmission of SARS-CoV-2 D614G, Alpha, Gamma, Delta, and Omicron variants in golden hamsters

**DOI:** 10.1038/s44298-025-00092-2

**Published:** 2025-02-24

**Authors:** Andra Banete, Bryan D. Griffin, Juan C. Corredor, Emily Chien, Lily Yip, Tarini N. A. Gunawardena, Kuganya Nirmalarajah, Jady Liang, Yaejin Lee, Alexander Leacy, Sara Pagliarani, Richard de Borja, Winfield Yim, Hunsang Lee, Yu Onodera, Patryk Aftanas, Patrick Budylowski, Sang Kyun Ahn, Yanlong Pei, Hong Ouyang, Laura Kent, Xinliu Angel Li, Mario A. Ostrowski, Robert A. Kozak, Sarah K. Wootton, Natasha Christie-Holmes, Scott D. Gray-Owen, Mikko Taipale, Jared T. Simpson, Finlay Maguire, Allison J. McGeer, Haibo Zhang, Leonardo Susta, Theo J. Moraes, Samira Mubareka

**Affiliations:** 1 Biological Sciences, Sunnybrook Research Institute, Toronto, ON Canada; 2 Program in Molecular Medicine, The Hospital for Sick Children, Toronto, ON Canada; 3 Program in Translational Medicine, Hospital for Sick Children, Toronto, ON Canada; 4 Keenan Research Centre for Biomedical Science, St. Michael’s Hospital, Unity Health Toronto, Toronto, ON Canada; 5 Department of Physiology, University of Toronto, Toronto, ON Canada; 6 Department of Pathobiology, University of Guelph, Guelph, ON Canada; 7 Ontario Institute for Cancer Research, Toronto, ON Canada; 8 Donnelly Centre for Cellular and Biomolecular Research, University of Toronto, Toronto, ON Canada; 9 Department of Emergency and Critical Care Medicine, Faculty of Medicine, Yamagata University, Yamagata, Japan; 10 Shared Hospital Laboratory, Toronto, ON Canada; 11 Department of Medicine, University of Toronto, Toronto, ON Canada; 12 Institute of Medical Science, University of Toronto, Toronto, ON Canada; 13 Department of Molecular Genetics, University of Toronto, Toronto, ON Canada; 14 Division of Comparative Medicine, Faculty of Medicine, University of Toronto, Toronto, ON Canada; 15 Department of Microbiology, Sinai Health System, Toronto, ON Canada; 16 Department of Immunology, University of Toronto, Toronto, ON Canada; 17 Department of Laboratory Medicine and Pathobiology, University of Toronto, Toronto, ON Canada; 18 Toronto High Containment Facility, Temerty Faculty of Medicine, University of Toronto, Toronto, ON Canada; 19 Department of Computer Science, University of Toronto, Toronto, ON Canada; 20 Department of Community Health and Epidemiology, Faculty of Medicine Dalhousie University, Halifax, NS Canada; 21 Faculty of Computer Science, Dalhousie University, Halifax, NS Canada; 22 Interdepartmental Division of Critical Care Medicine, University of Toronto, Toronto, ON Canada; 23 Department of Anaesthesiology and Pain Medicine, University of Toronto, Toronto, ON Canada; 24 Division of Respiratory Medicine, Department of Pediatrics, Hospital for Sick Children, Toronto, ON Canada

**Keywords:** Pathogenesis, SARS-CoV-2, Viral transmission

## Abstract

Since the emergence of SARS-CoV-2 in humans, novel variants have evolved to become dominant circulating lineages. These include D614G (B.1 lineage), Alpha (B.1.1.7), Gamma (P.1), Delta (B.1.617.2), and Omicron BA.1 (B.1.1.529) and BA.2 (B.1.1.529.2) viruses. Here, we compared the viral replication, pathogenesis, and transmissibility of these variants. Replication kinetics and innate immune response against the viruses were tested in ex vivo human nasal epithelial cells (HNEC) and induced pluripotent stem cell-derived lung organoids (IPSC-LOs), and the golden hamster model was employed to test pathogenicity and potential for transmission by the respiratory route. Delta, BA.1, and BA.2 viruses replicated more efficiently, and outcompeted D614G, Alpha, and Gamma viruses in an HNEC competition assay. BA.1 and BA.2 viruses, however, replicated poorly in IPSC-LOs compared to other variants. Moreover, BA.2 virus infection significantly increased secretion of IFN-λ1, IFN-λ2, IFN-λ3, IL-6, and IL-1RA in HNECs relative to D614G infection, but not in IPSC-LOs. The BA.1 and BA.2 viruses replicated less effectively in hamster lungs compared to the other variants; and while the Gamma virus reached titers comparable to D614G and Delta viruses, it caused greater lung pathology. Lastly, the Gamma and Delta variants transmitted more efficiently by the respiratory route compared to the other viruses, while BA.1 and BA.2 viruses transmitted less efficiently. These findings demonstrate the ongoing utility of experimental risk assessment as SARS-CoV-2 variants continue to evolve.

## Introduction

The global pandemic resulting from the emergence of the severe acute respiratory syndrome coronavirus 2 (SARS-CoV-2) *Betacoronavirus*, the causative agent of coronavirus disease 2019 (COVID-19), has resulted in hundreds of millions of confirmed infections worldwide, and greater than 7 million deaths^1^. SARS-CoV-2 infection often results in asymptomatic infection or mild illness, but can also lead to severe disease including respiratory failure and death, particularly in the elderly and individuals with predisposing medical conditions^2–4^. SARS-CoV-2 infection can also result in the post-acute infection syndromes collectively known as long COVID following both mild and severe infection^5^.

SARS-CoV-2 preferentially targets the epithelial cells of the upper and lower respiratory tract, although other tissues may become infected during severe COVID-19^6–8^. Attachment and viral entry into host cells of SARS-CoV-2 is mediated in large part by binding of the spike (S) protein to angiotensin-converting enzyme 2 (ACE2), a SARS-CoV-2 host cell receptor, and entry is facilitated by transmembrane protease serine 2 (TMPRSS2), a serine protease^9^. ACE2 and TMPRSS2 are present on various cell types, including ciliated nasal, bronchial, and bronchiolar epithelial cells, goblet cells, and pneumocytes, amongst others^10–12^. As SARS-CoV-2 continues to circulate among pre-exposed and/or vaccinated populations, new variants have rapidly emerged with altered pathogenicity, transmission potential, and immune evasion properties. The amino acid substitution D614G in the spike protein emerged in January 2020 and persisted in circulating lineages^13,14^. Several different variants with novel mutations subsequently emerged thereafter, including Alpha (B.1.1.7)^15^, Beta (B.1.351)^16^, Gamma (P.1)^17^, Delta (B.1.617.2)^18^, Omicron BA.1 parental (B.1.1.529)^19^, and the Omicron BA.2 sub-lineage (B.1.1.529.2) with associated recombinants^20^, which emerged in September 2020, October 2020, November 2020, March 2021, November 2021, and Jan 2022, respectively. Novel viruses were designated as ‘variants of concern’ (VOCs) by the World Health Organisation when associated with an increased transmission, more severe disease, a significant reduction in antibody neutralization, or impeded the effectiveness of molecular diagnostics, therapeutics, or vaccines. The variants examined in this study have been classified as VOCs, although some have been downgraded to ‘variants being monitored’ in some jurisdictions over the course of the pandemic.

Key amino acid changes in the S protein are among the characteristic features that in part define each variant, and these changes have been shown to affect the infectivity, pathogenicity, transmissibility, species tropism, and antigenicity of SARS-CoV-2. Highly relevant amino acid changes in the S protein include D614G (all VOCs)^21,22^, Del69-70 (Alpha, Omicron variants)^23,24^, N501Y (Alpha, Beta, Gamma, Omicron variants)^24,25^, L452R (Delta variant)^26^, E484K (Beta and Gamma variants)^24^, and P681R (Delta variant)^27^. Compared to the original emergent SARS-CoV-2 isolate, Wuhan/Hu-1/2019, the BA.1 and BA.2 VOCs have 36^28^ and 31^29^ amino acid substitutions in the S protein, respectively. Although the BA.1 and BA.2 variants share 20 of these substitutions, 11 amino acid changes are unique to BA.2^28,29^. Moreover, other studies have reported that BA.1 and BA.2 variants show reduced serum neutralizing antibodies induced by vaccination and viral infection, and reduced efficacy of therapeutic monoclonal antibodies^30–33^. Further, several studies indicate that the BA.2 variant is more transmissible in humans than the BA.1 variant^34,35^.

SARS-CoV-2 is known to transmit by direct and indirect contact, and by the respiratory route^36–39^. Hamster transmission studies, employing direct contact, fomite, and respiratory exposures have been used to study the transmissibility of D614G^21,22,40–42^, Alpha^25,40,43,44^, Delta^44,45–47^, and Omicron^42,46,48^ variants, as they have emerged. To our knowledge, there have been no hamster transmission studies for the Gamma variant. The SARS-CoV-2 Omicron lineage has shown variable capacity to replicate and transmit in the golden hamster model. While one study reported enhanced efficiency for non-contact (respiratory) transmission of Omicron BA.1 virus relative to Delta virus in hamsters^46^, the majority of studies have shown none, or significantly reduced transmission of Omicron BA.1^42,48^ and BA.2 viruses in hamsters^42,49^. The recombinant VOC XBB1.5 showed much greater transmissibility by the respiratory route in golden hamsters compared to BA.1 and BA.2^49^.

Here, we examine the replication kinetics and innate immune response of the D614G, Alpha, Gamma, Delta, and Omicron BA.1 and BA.2 variants in ex vivo human nasal epithelial cells (HNECs) and lung organoids. We further examine the pathogenesis of these variants in the established golden hamster model of SARS-CoV-2 infection and evaluate the capacity of these variants to transmit via respiratory exposure with directional airflow.

## Methods

### Ethics statement

The experiments described here were carried out at Sinai Health, St. Michael’s Hospital, Sunnybrook Research Institute, the Hospital for Sick Children, and the University of Toronto. Primary HNEC culture work was approved by the Research Ethics Board of the Hospital for Sick Children and St Michael’s Hospital (REB 1000044783 8 May 2014 and 1000061106 for cell collections and 1000071125 for COVID-19 related work), Toronto, ON, Canada; informed consent was obtained from all subjects that donated nasal cells for study. All animal experiments were carried out under the Animal User Protocol AUP# 20012669, approved by the Animal Care Committee at the University of Toronto in accordance with the guidelines provided by the Canadian Council on Animal Care. Animal procedures were carried out under inhalation anesthesia using isoflurane. All infectious work was performed under biosafety containment level 3 (CL-3) conditions at the Toronto High Containment Facility, Temerty Faculty of Medicine, University of Toronto.

### Cells

Vero E6 African green monkey (*Chlorocebus sp*.)-derived kidney epithelial cells (ATCC # CRL1586); Vero E6 cells modified to include cellular transmembrane serine protease II (TMPRSS2) (Vero E6-TMPRSS2^OE^); or Vero E6 with TMPRSS2 expressed and cathepsin L (CTSL) knocked out (Vero E6-TMPRSS2^OE^/CTSL^KO^)^50^ were cultured in Dulbecco’s Minimal Essential Medium (DMEM) (Wisent Bioproducts) supplemented with 5–10% Fetal Bovine Serum (FBS, Wisent Bioproducts) and L-glutamine. Vero-TMPRSS2 and Vero-TMPRSS2-CTSL^KO^ cells were passaged in the presence of 5 μg/ml of blasticidin for TMPRSS2 maintenance until seeding cells for the final infection. Calu3 cells (ATCC) were cultured in Minimal Essential Medium (MEM) (Wisent Bioproducts), supplemented with 5–10% Fetal Bovine Serum (Wisent Bioproducts) and L-glutamine. Cells were cultured at 37 °C with 5% CO_2_. Continuous cell lines were screened for mycoplasma prior to use; however, Vero and Calu3 cells were not authenticated prior to use and the sex of the cells is unknown.

HNECs were obtained from healthy donors and maintained in an air-liquid interface (ALI) for more than 28 days as previously described^51–53^. Briefly, HNECs frozen at passage number 1 were thawed and 3 × 10^5^ cells were seeded on a collagen-coated T75 flask (PneumaCult EX; StemCell Tech) at 37 °C with 5% CO_2._ Upon reaching 80% confluency the cells were passaged, and passage 3 cells were seeded onto collagen-coated transwells (6.5 mm diameter, 0.4 µm pore size; Corning) at a density of 1 × 10^5^ cells per transwell insert. HNECs were maintained in PneumaCult Ex Plus media until complete confluency was obtained and then the media was changed to a differentiation media (PneumaCult ALI; StemCell Technologies) at ALI. Prior to infection and at 0 dpi, media was changed every other day in the basolateral compartment. The age and sex of the donors was blinded to the investigators.

Human lung organoids were generated using a stepwise directed differentiation protocol with human induced pluripotent stem cells (iPSCs) (HDF-mRNA), provided by Drs. Snoeck and Chen^54^. Initially, undifferentiated iPSCs were induced to differentiate into the definitive endoderm lineage by exposure to saturating levels of Activin A. The embryoid bodies committed to the definitive endoderm were then patterned to the anterior foregut endoderm by sequential inhibition of transforming growth factor beta (TGFβ) and bone morphogenic protein signalling. Following this, the endodermal cells were ventralized, after which adherent cells were adapted to three-dimensional suspension culture. The cells were then promoted to the lung fate with a combination of growth and signaling factors, including the Wnt-agonist CHIR-99021, fibroblast growth factors, and retinoic acid. The lung organoids were maintained in lung organoid culture media (serum-free differentiation media supplemented with lung maturation factors) for 45 days, allowing them to reach maturity, expressing epithelial cell adhesion molecule (EpCAM) and surfactant protein C-Cre (SPC-Cre), before use.

### Virus isolation, propagation, and sequence verification

SARS-CoV-2 isolates, D614G (SG5, B.1 lineage), Alpha (SBA, B.1.1.7 lineage), Gamma (SBG, P.1 lineage), Delta (SBD, B.1.617.2), Omicron BA.1 (SBO1, B.1.1.529 lineage), and Omicron BA.2 (SBO2, B.1.1.529.2 lineage) were isolated from human oropharyngeal swab samples and propagated on either of three cells lines: Vero E6; Vero E6-TMPRSS2^OE^, Vero E6-TMPRSS2^OE^/CTSL^KO^. Briefly, patient mid-turbinate swab samples that had been frozen in virus transport medium were thawed, vortexed for 10 s, then centrifuged at maximum rpm for 5 min in a microcentrifuge. A total of 250 μL of virus supernatant from each cryovial was combined with 16 μg/ml TPCK trypsin and 2x antibiotic/antimycotic/penicillin/streptomycin (a/a/p/s) and was for some samples topped up to 250 μL with DMEM containing 0.1% BSA. This inoculant was then added to a 6 well plate well of Vero E6 (DG14G, Alpha, Gamma viruses) and Vero E6 TMPRSS2^OE^/CTSL^KO^ (Delta, Omicron BA.1, and Omicron BA.2 viruses) and incubated at 37 °C for 1 h with occasional rocking. After removing the inoculum, the medium was replaced with 2 ml of DMEM supplemented with 2% FBS, 1x L-glut, and 2x (a/a/p/s) (D2 medium), plus 6 μg/ml TPCK-trypsin. Some clinical samples were topped up to 1.5 ml with DMEM medium supplemented with 0.1% BSA and filtered with a Costar Spin-X 0.45 μm spin column (Corning) prior to adsorption to remove bacterial and fungal contamination. Cultures were monitored daily for cytopathic effect (CPE) and harvested when CPE became visible for greater than 50% of the monolayer or at 2–3 dpi, depending on the variant. Viral stocks were expanded in the same cells used for isolation and passage one (BA.1) or passage two (P2, all other VOCs) stocks were retained for downstream work and stored at –80 °C. For each SARS-CoV-2 variant stock, total RNA was extracted from cell culture supernatants using the QIAmp Viral RNA Mini Kit (Qiagen), according to the manufacturer’s instructions. The resulting SARS-CoV-2 stocks underwent paired-end (2 × 150 bp) whole genome sequencing (WGS) using the ARCTIC V4 primer pool on an Illumina MiniSeq Sequencing System, and data was then processed for quality control, mapped to the MN908947.3 reference genome, primer sequences trimmed, variants called, and lineages (PANGO v4.3.1) inferred using the SIGNAL v1.6.1 workflow^55^. This was used to confirm virus lineage and mutation profile after variant passaging in cell culture. Sequencing was carried out to identify the viral lineage of each isolate and to confirm single nucleotide polymorphisms (SNPs) that were not present above 5% in the initial swab sample were not introduced (ie. the propagated virus stock was not meaningfully different than the initial input isolate virus), and to ensure that the furin cleavage site and furin-proximal regions within the spike protein were maintained in greater than 90% of the sequenced virus population. The genomic sequence of the SARS-CoV-2 SG5, SBA, SBG, SBD, SBO1, SBO2 isolates were submitted to the GISAID database. Virus stocks were titered using Vero E6-TMPRSS2^OE^/CTSL^KO^ by the standard median tissue culture infectious dose (TCID_50_) method.

### Ex vivo cell infections

Ex vivo differentiated human induced pluripotent stem cell-derived lung organoids (IPSC-LO) were added to each well of a 12 well plate (average of 5 × 10^6^ cells/well). Following physical disruption of the lung organoids by gentle pipetting, lung organoids were washed three times with 500 μL of PBS and were then infected with the respective SARS-CoV-2 variants (DG14G, Alpha, Gamma, Delta, Omicron BA1, and Omicron BA2 viruses) at an MOI of 0.1 TCID_50_/cell in a volume of 500 μL. Virus was adsorbed at 37 °C for 1 h with manual shaking every 15 min, after which the inoculum was removed and IPSC-LOs were washed and maintained in lung maturation medium. At each time point, 20% of the supernatant was removed (200 μL) for downstream assays, and was replaced with fresh medium. HNECs were seeded on to ALI transwells (average of 2 × 10^6^ cells/transwell) and maintained in a 24 well plate wells with regular removal of mucus (100 μL PBS wash with incubation at 37 °C) and replacement of basal medium with fresh ALI media every other day for at least one month, and after well matured with visible cilia throughout the transwell, as previously described^52^. Cells were infected with 200 μL of virus at an MOI of 0.1 and incubated for 1 h with gentle rocking every 15 min, after which the apical side of the transwell was washed four times with 200 μL PBS at 37 °C. For time point collections, cells were washed twice with PBS, then 200 μL of PBS was added to the apical side of the ALI and incubated for 15 min at 37 °C before collection and storage at –80 °C.

### SARS-CoV-2 variant competition assay

HNECs were seeded and infected as described above at an MOI of 0.1 with the exception that the inoculum consisted of a ratio of two different SARS-CoV-2 variants at a 1:10 ratio by TCID_50_ and maintained in the ALI condition. At the evaluated time points, cells were washed twice with PBS, then 200 μL was added to the apical side of the transwell and incubated for 15 min, then harvested and stored at –80 °C. Samples were later thawed and RNA was extracted from the apical washes using the QIAmp Viral RNA Mini Kit (Qiagen), according to the manufacturer’s instructions. WGS was then performed using the ARCTIC V4 primer pool. Paired-end (2 × 150 bp) sequencing was performed on an Illumina MiniSeq. Variant ratios were determined using freyja (https://github.com/andersen-lab/Freyja)^56^. All samples were initially processed by the CanCOGen-VirusSeq analysis pipeline (https://github.com/jts/ncov2019-artic-nf) to remove sequencing adapters, map reads to the SARS-CoV-2 reference genome (accession MN908947.3), remove amplification primers and identify single nucleotide variants (SNVs). These results were used to derive SNV presence/absence barcodes for the six input viruses (D614G, Alpha, Gamma, Delta, Omicron BA.1, Omicron BA.2). These barcodes were used to estimate variant proportions from the mapped reads using the ‘freyja variants’ and ‘freyja demix’ commands with default parameters.

### Multiplex analysis of human cytokines, chemokines, and growth factors

Basolateral medium was sampled from infected HNECs and supernatants were sampled from infected IPSC-LOs at 3 days post infection (basolateral medium was not changed after 0 dpi). 50 μL of sample were inactivated with TritonX-100 in FBS to a final concentration of 1%, and incubated for 1 h at RT. Samples were passed through a 0.22 μm filter. Cytokine analysis was performed by Eve Technologies Corp. (Calgary, Alberta) using the Luminex 200 system (Luminex, Austin, TX, USA) and the Human Cytokine Proinflammatory Focused 15-Plex Discovery Assay Array (HDF15) and Human Interferon 9-Plex Discovery Assay (Millipore Sigma, Burlington, Massachusetts, USA) according to the manufacturer’s protocol. The proinflammatory cytokine 15-plex tests for GM-CSF, IFNγ, IL-1β, IL-1Ra, IL-2, IL-4, IL-5, IL-6, IL-8, IL-10, IL-12p40, IL-12p70, IL-13, MCP-1 and TNF-α, with assay sensitivities ranging from 0.14 to 5.39 pg/mL. The interferon 9-plex tests for IFNα2, IFNβ, IFNε, IFNλ1, IFNλ2, IFNλ3, IFNω, IFNγ, and IFNγR1, with assay sensitivities ranging from 1.27 to 100.2 pg/mL. Sensitivity values for individual analytes are available in the MilliporeSigma MILLIPLEX® MAP protocol.

### Hamster challenge experiments

Six-to-twelve-week-old male or female Syrian golden hamsters used for these experiments were obtained from Charles River and randomly assigned to the different groups. All animals were acclimated for at least one week prior to the experiments. Groups consisting of equal numbers of male and female hamsters were anaesthetized and exposed to 10^4^ TCID_50_ SARS-CoV-2 or mock infected (DMEM) by an intranasal route of inoculation in a volume of 20 μL with the inoculum distributed evenly into both nares. Animals were weighed and monitored daily for clinical signs of disease and scoring, and were either necropsied at 3 dpi, 7 dpi, or 21 dpi, as indicated.

### Measurement of viral burden in tissues

Viral titres in tissues of infected animals were measured by standard TCID_50_ assays. Following necropsy, lung and nasal turbinate samples were collected and frozen at –80 °C for storage. Tissue samples were thawed, weighed, and placed in 1 ml DMEM supplemented with 1× L-glutamine, 2x penicillin-streptomycin, and 1% FBS, then homogenized using 5 mm stainless steel beads in an Omni Bead Ruptor Elite Tissue Homogenizer at 4 m/s for 90 s. Homogenates were centrifuged for 6 min at 1500 × *g*, then tenfold serially diluted in DMEM supplemented with 1x L-glutamine, penicillin-streptomycin, and 1% FBS. Homogenate dilutions were added to confluent VeroE6-TMPRSS2^OE^/CTSL^KO^ cells in triplicate wells, and cytopathic effect was recorded at 5 dpi. The TCID_50_ values were calculated by the modified Reed and Muench method^57^.

To examine viral RNA in tissues, nasal turbinate and lung samples were harvested and stored in RNAlater, according to the manufacturer’s directions. Total RNA was extracted using the RNAeasy Plus Mini Kit (Qiagen), according to the manufacturer’s instructions. RT-qPCR detection of SARS-CoV-2 was performed on a Rotorgene Q real-time PCR system (Qiagen) using a Luna Universal Probe One-Step RT-qPCR kit (New England Biolabs) and primers specific for the SARS-CoV-2 E gene as per the diagnostic protocol recommended by the World Health Organization (Forward – ACAGGTACGTTAATAGTTAATAGCGT; Reverse – ATATTGCAGCAGTACGCACACA; Probe – FAM-ACACTAGCCATCCTTACTGCGCTTCG-BHQ-1) (Supplementary Table 1). For the viral RT-qPCR assay, 7 μL of extracted RNA was assayed in duplicate wells of a 96 well plate. Oligonucleotide concentrations were 400 nM for the primers and 200 nM for the probe. RT-qPCR cycling stages were as follows: reverse transcription (60 °C for 10 min), denaturation (95 °C for 2 min), followed by amplification (44 cycles of 95 °C for 10 s and 60 °C for 15 s).

### Detection of virus in swab samples

Animals were anesthetized and oropharyngeal swab samples (FLOQSwabs flocked swabs, Copan) were collected and stored in 1 ml DMEM + 2% penicillin-streptomycin at –80 °C. Prior to quantification by TCID_50_ assay, cryovials containing swab samples and swabs were briefly vortexed for 10 s and centrifuged at 2400 rpm for 3 min in a microcentrifuge. Viral RNA was extracted using a QIAmp Viral RNA Mini Kit (Qiagen), according to the manufacturer’s instructions and RT-qPCR was performed as above.

### Hamster immune gene analysis

Total RNA from tissue was extracted as described above with the exception that genomic DNA was removed by passing samples through a genomic DNA eliminator column (Qiagen). These samples were used to determine host mRNA expression of select immune genes, including *IL-1β, IL-6, TNF-α, IL-2, IL-4, IL-6, IL-10, IRF1, IFNλ*, and *IFNγ*. Gene expression was quantified using a previously designed primer-probe set with *RPL18* housekeeping gene as an internal control^58,59^. Briefly, RT-qPCR was carried out using a Luna Universal Probe One-Step RT-qPCR kit (New England Biolabs) using 3 μL of extracted RNA for RT-qPCR on the QuantStudio™ 3 Real-Time PCR System (Thermo Fisher Scientific). Primers and probes are listed in Supplementary Table 1. RT-qPCR cycling stages were as follows: incubation (25 °C for 2 min), reverse transcription (60 °C for 10 min), denaturation (95 °C for 1 min), followed by amplification (44 cycles of 95 °C for 10 s and 60 °C for 30 s). Following RT-qPCR, the Delta-Delta Ct method was used to calculate fold change in gene expression compared to *RPL18*.

### Histopathology and immunohistochemistry

Formalin-fixed lung and nasal turbinate tissues were routinely embedded in paraffin, cut in 5 μm thick sections, and stained with hematoxylin and eosin (H&E). After fixation, nasal turbinates were decalcified for ~72 h (Cal-Ex® II, Fisher Chemical®). For each hamster, the final microscopic lesion score was calculated separately for nasal turbinates and lung tissues, as previously described^60^. For the nasal turbinates, the nominal categories “inflammatory lesions”, “exudate on the surface of the mucosa”, and “epithelial damage” were binarily graded (0, absence; 1 presence) and summed to reach a final score (range 0–3) for each hamster. For the lungs, the final score considered 11 nominal categories and the extent of lung area affected. The 11 nominal categories were graded as present (1) or absent (0), with the exception of “edema / hemorrhage / fibrin” in alveoli (2, present; 0, absent), “severe vascular changes” (endothelialitis; 2, present; 0, absent)^61^, and “macrophages with pigment”(0.5, present; 0, absent), which were weighted differently to represent different contributions to the lesions’ severity. The area grade was semi-quantitatively divided into five tiers of percentage tissue involvement: 0–5% (grade = 0), 6–15% (1), 16–40% (2), 41–60% (3), and 61–100% (4). The nominal grades were summed (0–12.5) and multiplied by the area grade, for a final score ranging between 0 and 50.

Presence of virus in the hamster lung tissue was evaluated by immunohistochemistry (IHC) against SARS-CoV-2 nucleoprotein (N). Briefly, antigens were unmasked by boiling slides in Target Retrieval Solution (pH 9; S2367; Dako/Agilent Technologies Canada Inc), and tissues were then blocked with a Dual Endogenous Enzyme Block (S2003; Dako/Agilent Technologies Canada Inc) and Protein Block Serum-Free (X0909; Dako/Agilent Technologies Canada Inc) before incubation with a monoclonal anti-SARS-CoV-2 nucleoprotein (MAB10474; R&D Systems) diluted to 1.25 μg/mL in Antibody Diluent (S0809; Dako/Agilent Technologies Canada Inc). Following primary incubation, sections were incubated with goat anti-mouse/rabbit peroxidase-linked polymer detection system (K4061; EnVision+; Dako/Agilent Technologies Canada Inc) and Liquid DAB+ Substrate Chromogen System (K3468; Dako/Agilent Technologies Canada Inc). Colorimetric reaction was developed for ~5 min, and slides were then counterstained with hematoxylin before being coverslipped. The magnitude of immunohistochemical reactivity was graded by tallying presence (1) or absence (0) of labeling in the airways or alveoli (separately), which were summed (range, 0–2) and multiplied by the same area grade reported above. The final IHC score for each lung ranged between 0 and 8.

### Measurement of antibodies

Pre-coated 96-well flat bottom plates from a Human SARS-CoV-2 Spike (Trimer) IgG ELISA kit (ThermoFisher Scientific, cat. # BMS2323) were used according to the kit instruction, except for substitution of the secondary antibody for the detection of hamster IgG antibodies. Briefly, hamster serum was diluted to 1:100 final concentration. The secondary antibody was substituted with a goat-anti Syrian hamster IgG (H + L) secondary antibody conjugated to HRP (ThermoFisher Scientific, cat. # PA1-29626) at a 1:10,000 dilution.

### Hamster transmission studies

To evaluate the transmission potential of SARS-CoV-2 variants through the respiratory route, donor animals were inoculated under isoflurane anesthesia as described above. At 1 dpi, sex-matched naïve and donor hamsters (1:1 ratio) were transferred to clean transmission cages with an airflow permissive polycarbonate divider creating a separation of 9 cm between the animals with directional airflow (Supplementary Fig. 2) from donor to naïve using a HEPA filtered airflow unit. DietGel (ClearH2O) was provided to the hamsters during the cohousing period ad libitum. To minimize unintentional fomite transmission, a low-dust corn bedding product was used and the bottom 5 cm of the divider did not have aeration holes to prevent the transfer of bedding. Hamsters were monitored daily for weight changes and signs of disease. Oropharyngeal swabs were collected (under anesthesia) from donor and naïve hamsters on days 0, 1, 2, 4, and 6 after inoculation and exposure, respectively. After an exposure period of 7 days, animals were single-housed in separate cages and monitored daily for 14 d. For the adeno-associated virus transmission studies, six-to-twelve-week-old female and male (*n* = 2 donors and 2 recipients) Syrian golden hamsters (*Mesocricetus auratus*) were administered 10^11^ vector genomes (vg) of adeno-associated virus (AAV6.2FF) expressing hACE2 or a luciferase control by an intranasal route in a volume of 180 μL. After 14 days, donor hamsters were inoculated with 10^4^ TCID_50_ of Omicron BA.2 virus by a low volume inoculum (20 μL) intranasal route of administration and carried out as above.

### Data analysis

Results were analyzed and graphed using Prism 9 software (GraphPad Software). Statistical analyses were carried out as appropriate using ANOVA with multiple comparison correction. Researchers were not blinded for these experiments except the pathological analyses on hamster tissues were carried out in blinded groupings. Figure 6A was created in BioRender (https://BioRender.com/g01q595).

## Results

### In vitro and ex vivo characterization of SARS-CoV-2 Alpha, Gamma, Delta, and Omicron variants

**Fig. 1:**
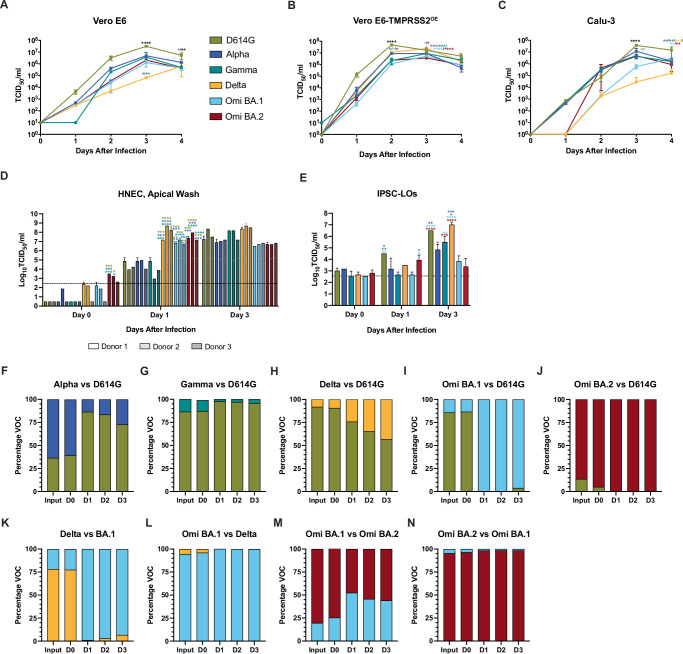
In vitro and ex vivo SARS-CoV-2 replication kinetics and variant competition assays. In vitro and in vivo characterization of the SARS-CoV-2 variants, D614G (gold), Alpha (dark blue), Gamma (teal), Delta (orange), Omicron BA.1 (light blue), Omicron BA.2 (maroon) viruses. Where statistics are indicated two-way ANOVA was carried out: *p ≤ 0.05, **p ≤ 0.01 ***p ≤ 0.001, ****p ≤ 0.0001, and the asterisk color indicates the comparator group whereby black indicates a statistically significant difference compared to all the other groups individually. Growth curves of SARS-CoV-2 variants in **A** Vero E6, **B** Vero E6-TMPRSS2^OE^, and **C** Calu-3 cell lines inoculated with an MOI = 0.01 TCID_50_ with two biological replicates (*n* = 2). Squares represent means and error bars indicate s.d. **D** Replication kinetics of SARS-CoV-2 variants in ex vivo differentiated human nasal airway epithelial cells (HNEC). HNECs derived from three donors were mock-infected or infected with the indicated SARS-CoV-2 variant at MOI = 0.1 TCID_50_. Donor one, two, and three are indicated by empty, dotted, and diagonal hatched bars, respectively. For the indicated times after infection for each SARS-CoV-2 variant, one, two, and three days-post infection (dpi), infectious titers of apical washes are shown. Bars represent the mean ± s.d. Two biological replicates (*n* = 2) were performed. The limit of detection (LOD) is indicated by a dashed line. **E** Replication of SARS-CoV-2 variants in ex vivo differentiated human induced pluripotent stem cell-derived lung organoids (IPSC-LO). IPSC-LOs were mock-infected or infected with the indicated SARS-CoV-2 variant at MOI = 0.1. Viral titers in cell supernatants were determined at the indicated times after infection (one, two, and three dpi). Bars represent the mean ± s.d. and include two biological replicates (*n* = 2). The LOD is indicated by a dashed line. **F**–**N** SARS-CoV-2 variant competition assay, where HNECs were inoculated with a mixture of D614G and Alpha (**F**), D614G and Gamma (**G**), D614G and Delta (**H**), D614G and Omicron BA.1 (**I**), D614G and Omicron BA.2 (**J**), Delta and Omicron BA.1 (**K**), Omicron BA.1 and Delta (**L**), Omicron BA.1 and BA.2 (**M**), or Omicron BA.2 and BA.1 (**N**) at a ratio of 10:1 of infectious virus (MOI = 1: MOI = 0.1). Total RNA was extracted from initial virus input and the apical washes obtained at the indicated times after infection (one, two, and three days post-infection) and subjected to whole genome sequencing using the ARCTIC V4 SARS-CoV-2 primer pool to determine the ratio of genome copies of each variant within the samples (distinguished bioinformatically by single nucleotide polymorphisms).

In order to examine replication kinetics, representative isolates of SARS-CoV-2 lineage D614G, Alpha, Gamma, Delta, Omicron BA.1, and Omicron BA.2 variants were compared in Vero E6 (Fig. 1A), Vero E6-TMPRSS2^OE^ (Fig. 1B), and Calu-3, a human lung adenocarcinoma-derived epithelial cell line (Fig. 1C) at a multiplicity of infection (MOI) of 0.01. In Vero E6 cells, the infectious titers of D614G virus in cell supernatants were consistently elevated compared to the other variants, reaching a peak mean titer of 7.5 tissue culture infectious dose 50% (TCID_50_) /ml (log_10_) versus 6.7 (Alpha), 6.6 (Gamma), 4.8 (Delta), 6.1 (BA.1), and 6.3 (BA.2) log_10_TCID_50_/ml, and were significantly different at 3 days post infection (dpi) (*p* < 0.0001) and 4 dpi (*p* < 0.005 (Gamma, Delta, Omicron BA.1. and Omicron BA.2) and *p* < 0.0222 (Alpha)). A similar trend was observed in Vero E6-TMPRSS2^OE^, where D614G virus-infected cell supernatants bore elevated titers relative to all the other variants at 2 dpi (*p* < 0.0001), and the titer of D614G supernatants remained elevated at 3 dpi relative to Omicron BA.1 and BA.2 viruses (*p* < 0.0065 and 0.0014, respectively).

D614G virus reached a peak titer of 7.7 log_10_ TCID_50_/ml at 2 dpi, whereas the other variants reached their mean peak a day later (6.9 (Alpha), 6.9 (Gamma), 7.4 (Delta), 6.7 (BA.1), and 6.6 log_10_ TCID_50_/ml (BA.2)) when D614G virus titers had decreased to 7.2 log_10_ TCID_50_/ml. In contrast to infection in Vero E6, Delta virus titers in Vero E6-TMPRSS2^OE^ were elevated relative to Gamma, BA.1, and BA.2 at 2 dpi (*p* < 0.05) and elevated relative to Alpha, Gamma, BA.1, and BA.2 at 3 dpi (*p* < 0.001 for Alpha and Gamma, and *p* < 0.0001 for BA.1 and BA.2). Lastly, in Calu-3 cells viral titers were not significantly different until 3 dpi when D614G titers reached a peak mean titer of 7.6 log_10_ TCID_50_ /ml versus 7.1 (Alpha), 6.6 TCID_50_ (Gamma), 4.4 TCID_50_ (Delta), 5.8 TCID_50_ (BA.1), 6.7 log_10_ TCID_50_/ml (BA.2) and were significantly elevated compared to all other variants (*p* < 0.0001).

In order to examine replication kinetics in more clinically-relevant models, the six SARS-CoV-2 variants were compared in ex vivo primary human nasal epithelial (HNEC) derived from airway brush biopsies from three donors^51^. HNECs were inoculated at an MOI of 0.1 TCID_50_ in duplicate wells, and daily washes of the apical surface of the ALI were harvested to quantify the virus titers by the standard TCID_50_ assay (Fig. 1D). Thus, each bar represents the mean titer of released virus since the previous wash (24 h). At 0 dpi, immediately following virus adsorption and washes, the mean titers for D614G, Alpha, Gamma, Delta, and BA.1 viruses were below the limit of detection (LOD) for the assay (2.4 log_10_ TCID_50_/ml), while the mean BA.2 virus titers ranged between 2.6 and 3.5 log10 TCID_50_/ml and were significantly elevated compared the mean titers for D614G, Alpha, and Gamma viruses for donor 1 (*p* < 0.001) and the mean titers of D614G and Gamma viruses for donor 2 (*p* < 0.05). At 1 dpi, the mean titers of D614G, Alpha, and Gamma viruses did not differ statistically from one another (ranging from 3.0 to 4.9 log10 TCID_50_/ml); however, they were significantly lower than Delta, Omicron BA.1 and Omicron BA.2 viruses from all three donors (*p* < 0.05 for all variant donor pairings). At 3 dpi, the apical wash titers of the D614G, Alpha, and Gamma viruses had increased to reach a comparable titer to Delta, Omicron BA.1, and Omicron BA.2.

Next, induced pluripotent stem cell-derived lung organoids (IPSC-LO) derived from matured human induced pluripotent stem cells (iPSCs)^62–64^ were infected at an MOI of 0.1 TCID_50_, and supernatants were sampled daily and titered by standard TCID_50_ assay (Fig. 1E). At 1 dpi, D614G virus titers reached 4.5 log_10_ TCID_50_/ml and were significantly higher compared to Alpha (3.2 log_10_ TCID_50_/ml), Gamma (2.7 log_10_ TCID_50_/ml), and BA.1 (2.7 log_10_ TCID_50_/ml) viruses (*p* = 0.0343, 0.0024, and 0.0024, respectively), while the BA.2 virus titers reached 3.9 TCID_50_/ml and were significantly higher compared to Gamma (2.7 log_10_ TCID_50_/ml) and BA.1 (2.7 log_10_ TCID_50_/ml) viruses (*p* = 0.0469). At 3 dpi BA.1 virus titers peaked at 3.8 log_10_ TCID_50_/ml and were significantly lower compared to D614G (6.5 log_10_ TCID_50_/ml), Gamma (5.5 log_10_ TCID_50_/ml), and Delta (7.0 log_10_ TCID50/ml) (*p*, 0.001, *p* = 0.0059, and *p* < 0.001, respectively). At the same time point, BA.2 virus titers reached 3.4 log_10_ TCID_50_/ml and were significantly lower than D614G (6.5 log_10_ TCID_50_/ml), Alpha (4.8 log_10_ TCID_50_/ml), Gamma (5.5 log_10_ TCID_50_/ml), and Delta (7.0 log_10_ TCID_50_/ml) viruses (*p* < 0.001, *p* = 0.0179, *p* = 0.0005, and p < 0.001), and the titers of the D614G and Delta viruses were significantly elevated relative to Alpha virus (*p* = 0.0059 and *p* = 0.0004).

To assess differences in replicative fitness in HNECs, single transwell inserts were inoculated with combinations of two different SARS-CoV-2 variants simultaneously inoculated at a target ratio 1:10 TCID_50_ in a competition assay. Apical washes were obtained daily to quantify the genomic copies (independent of infectivity) of each virus variant released over time (Fig. 1F–N). D614G virus demonstrated enhanced replication compared to Alpha (Fig. 1F) and Gamma (Fig. 1G); however, this trend was reversed for D614G virus relative to Delta (Fig. 1H), BA.1 (Fig. 1I), and BA.2 (Fig. 1J) viruses. The BA.1 and BA.2 viruses rapidly outcompeted D614G by 1 dpi (Fig. 1I, J), noting that the input genomic quantity of BA.2 was lower than D614G. Omicron BA.1 outcompeted Delta virus (Fig. 1K, L). It is important to note that differences in RNA copies:infectious virus ratios for the different variants may have influenced the outcomes and that the competition assays do not depict quantified infectious virus, but viral genome copies.

### Immune response of human-derived primary cells to VOC infection

**Fig. 2:**
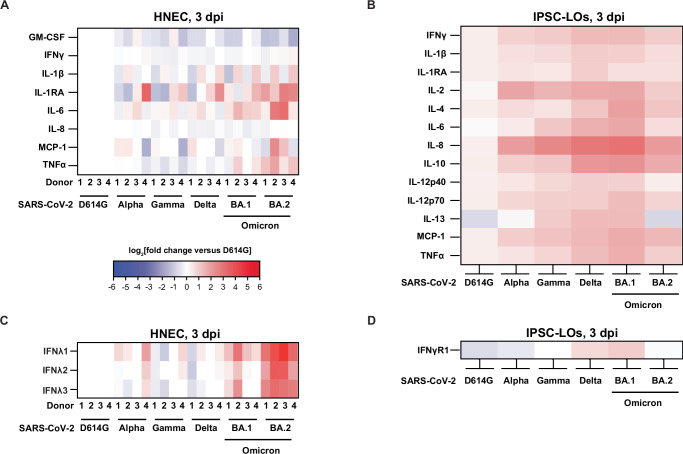
Immune response of human-derived primary cells to SARS-CoV-2 infection. Heatmaps illustrating the cytokine levels detected in SARS-CoV-2 D614G, Alpha, Gamma, Delta, Omicron BA.1, and Omicron BA.2 variant-infected HNEC basolateral medium samples (**A**/**C**) or IPSC-LO supernatants (**B**/**D**) at 3 days post-infection (dpi) shown as the log_2_(fold change) relative to the D614G virus-infected matched donor group (HNEC *n* = 4, two independent experiments; IPSC-LO, n = 6, three independent experiments (*n* = 2) combined data). The experiments depicted in panels **A** and **B** used the Luminex xMAP platform for multiplexed quantification of 15 human cytokines, chemokines, and growth factors using the Luminex 200 system. Fifteen markers were simultaneously measured in the samples using Eve Technologies’ Human Focused 15-Plex Discovery Assay (MilliporeSigma), according to the manufacturer’s protocol. The 15-plex consisted of GM-CSF, IFNγ, IL-1β, IL-1Ra, IL-2, IL-4, IL-5, IL-6, IL-8, IL-10, IL-12p40, IL-12p70, IL-13, MCP-1 and TNF-α. The experiments depicted in panels **C** and **D** used the Luminex xMAP platform for multiplexed quantification of nine human interferon cytokines. using the Luminex 200 system. Nine markers were simultaneously measured in the samples using Eve Technologies’ Human Interferon 9-Plex Discovery Assay (MilliporeSigma) according to the manufacturer’s protocol. The 9-plex consisted of IFNα2, IFNβ, IFNε, IFNλ1, IFNλ2, IFNλ3, IFNω, IFNγ, and IFNγR1. Individual sample values below the limit of detection (LOD) were replaced with the LOD value for that assay. Cytokines were excluded from depiction in the figure when the majority of samples were below the LOD for the assay, but the full dataset is provided in a Supplementary Table. TNFα tumor necrosis factor α, MCP-1 monocyte chemoattractant protein-1, IL interleukin, RA receptor antagonist, IFN interferon, GM-CSF granulocyte-macrophage colony-stimulating factor.

Interferons (IFNs) are the main first-line innate immune defense against viral infections, and IFNλ in particular has been shown to restrict SARS-CoV-2 infection in clinical trials^65^. To understand the cytokine/chemokine profile secreted by human-derived primary cells after SARS-CoV-2 infection, we collected the basolateral medium (HNECs) or growth medium (IPSC-LO) at 3 dpi for screening using a 15-plex proinflammatory focused array (GM-CSF, IFNγ, IL-1β, IL-1RA, IL-2, IL-4, IL-5, IL-6, IL-8, IL-10, IL-12p40, IL-12p70, IL-13, MCP-1, TNFα) and a 9-plex interferon array (IFNα2, IFNβ, IFNε, IFNγ Receptor 1, IFNλ1, IFNλ2, IFNλ3, IFNω); the complete data set, including analytes below the LOD for the assay are included in the Supplementary Tables 2 and 3. Where statistics are indicated, two-way ANOVA was carried out. Infection of HNECs resulted in moderately increased cytokine production, with small differences in protein expression of the assayed cytokines and chemokines detected between variants (Fig. 2A). Only IL-6 and IL-1RA were significantly higher in Omicron BA.2 virus-infected HNECs compared to D614G, Gamma, and Delta infections (IL-6 D614G vs BA.2 *p* = 0.0377, *p* = 0.0093; IL-1RA Delta vs BA.2 *p* = 0.0174, Gamma vs BA2 *p* = 0.0063, Delta vs BA.2 *p* = 0.0065) D614G (Fig. 2A). Although GM-CSF secretion was reduced in infection with all VOCs compared to D614G, it did not constitute a significant decrease. Significantly elevated concentrations of all IFNλ subtypes (IFNλ1, IFNλ2, and IFNλ3) were induced in response to BA.2 infection across all 4 donors (*p* < 0.01) and 2/4 donors in response to BA.1 infection (*p* < 0.05), which was not observed in response to infection with the other VOCs (Fig. 2C). In IPSC-LOs, compared to D614G, there were significantly higher levels of IL-2 (Alpha, Gamma, Delta, BA.1, *p* < 0.001) and IL-8 (all VOCs, with the highest levels observed in response to BA.1 infection, *p* < 0.0001), TNFα (Delta and BA.1 viruses, *p* = 0.0371 and *p* = 0.0015, respectively), MCP-1 (Delta, BA.1, and BA.2 viruses, *p* = 0.0144, 0.0015, and 0.0117), IL-6 (Delta, BA.1, and BA.2 viruses, *p* = 0.0483, 0.0003, <0.0001), and IL-12p70 (BA.1 virus, *p* = 0.0353) observed. Compared to D614G and BA.2 infection, IL-13 secretion was significantly upregulated with Gamma, Delta, and BA.1 infections (*p* < 0.0001). The anti-inflammatory cytokine IL-10 was significantly upregulated in Delta, BA.1, and BA.2 viruses compared to D614G (*p* < 0.0001, *p* < 0.0001, and *p* = 0.0014). No production of type I IFN was detected, and only moderate amounts of IFNγR1 were observed in response to Delta and BA.1 infection (Fig. 2D).

### SARS-CoV-2 infection of golden hamsters

**Fig. 3:**
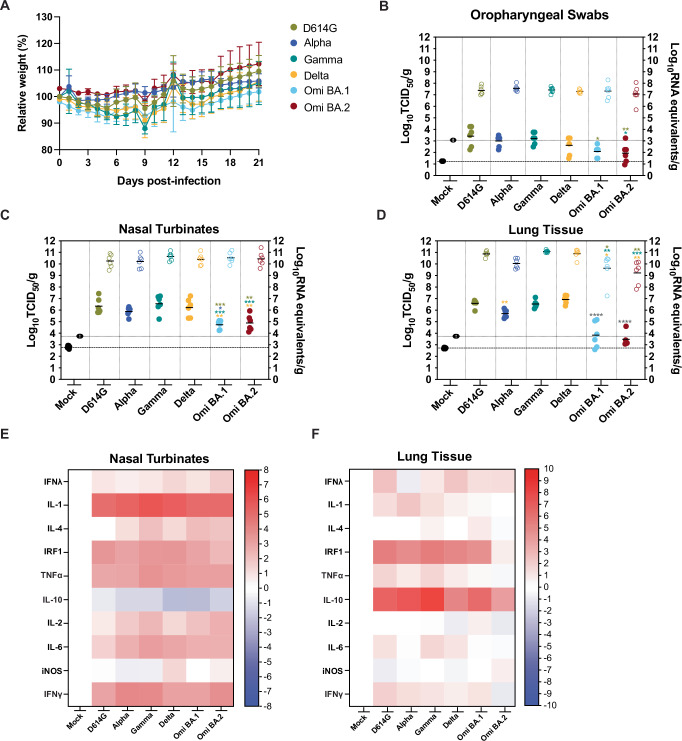
SARS-CoV-2 infection of golden hamsters. Six-to-twelve-week-old female and male golden hamsters (*Mesocricetus auratus*) were mock inoculated (black) or inoculated with 10^4^ TCID50 of each SARS-CoV-2 variant, D614G (gold), Alpha (dark blue), Gamma (teal), Delta (orange), Omicron BA.1 (light blue), and Omicron BA.2 (maroon) virus, using a low volume inoculum (20 µL) via the intranasal route of administration. **A** Animals were weighed daily up to 21 days post infection (dpi) (one experiments, *n* = 4). Circles indicate the mean ± s.e.m. infectious viral load (filled in shapes, left axis) and vRNA levels (empty shapes, right axis) were measured at 3 dpi in oropharyngeal swab samples (**B**), nasal turbinates (**C**), and lungs (**D**). Data were collected in a series of three experiments (*n* = 2 per group). Lines indicate means and error lines indicate s.d. Where statistics are indicated one-way ANOVA was carried out: *p ≤ 0.05, **p ≤ 0.01 *** *p* ≤ 0.001, **** *p* ≤ 0.0001 and the asterisk color indicates the comparator group with grey indicating a statistically significant difference compared to all the other groups individually, but excluding the mock-infected group. Heatmaps illustrating the cytokine transcript levels detected in SARS-CoV-2 D614G, Alpha, Gamma, Delta, Omicron BA.1, and Omicron BA.2 variant-infected hamster nasal turbinate (**E**) and lung (**F**) tissues.

To compare SARS-CoV-2 pathogenesis for the D614G, Alpha, Gamma, Delta, Omicron BA.1, and Omicron BA.2 viruses, groups of 4 six-to-twelve-week-old female (*n* = 2) and male (*n* = 2) Syrian golden hamsters (*Mesocricetus auratus*) were inoculated with 10^4^ TCID_50_ of each variant by a low-dose (20 μL) intranasal route of infection. Inoculation of hamsters with Alpha, Gamma, Delta, and Omicron BA.1 and BA.2 viruses resulted in productive infection. Hamsters were weighed daily for potential recovery until 21 dpi (Fig. 3A). In all the variant virus groups, the average hamster weight dropped at 2 dpi and increased again at 7 dpi with a transient drop at 9 dpi, and there were no statistically significant differences between the groups (*p* > 0.05). Notably, at 21 dpi at least one hamster per group following infection had failed to reach or exceed the initial starting weight. A separate set of animals was similarly infected (*n* = 3 female and *n* = 3 male), and the SARS-CoV-2 burden in the upper and lower respiratory tract was assessed by infectious titers (left axis, solid circles) and genomic copies of viral RNA (vRNA) (right axis, empty circles) at 3 dpi in oropharyngeal swab samples (Fig. 3B), nasal turbinates (Fig. 3C) and lungs (Fig. 3D). When statistics are indicated a one-way ANOVA was carried out. The SARS-CoV-2 vRNA detected in the oropharyngeal swab samples ranged from 5.7 to 8.3 log_10_ RNA equivalents/g (Fig. 3B) and did not differ significantly between any of the groups; however, the mean infectious titers of the D614G virus were elevated compared to the other groups and was significantly higher than those detected in the BA.1 and BA.2-infected animals (3.4 log_10_ TCID_50_/g versus 2.2 and 1.9, *p* = 0.0139 and 0.0052). Further, the oropharyngeal swabs from the Gamma virus-infected hamsters had significantly elevated mean infectious titers compared to the BA.2 group at 3 dpi (*p* = 0.0235). The quantity of vRNA in the nasal turbinates samples ranged from 9.5 to 11.4 log_10_ RNA equivalents/g with no significant differences detected between the groups (*p* > 0.05). The mean infectious titers of the BA.1 and BA.2 nasal turbinates were at least one log lower than the other inoculated groups. Mean BA.1 titers were significantly lower than D614G, Alpha, Gamma, and Delta virus groups (*p* = 0.0007, 0.0264, 0.0001, and 0.0019, respectively), and mean BA.2 titers were significantly lower than D614G, Gamma, and Delta virus groups (*p* = 0.0022, 0.0004, 0.0060, respectively). The SARS-CoV-2 mean vRNA detected in the lungs (Fig. 3D) was lower in the Alpha (10.0 log_10_ RNA equivalents/g), BA.1 (9.6 log_10_ RNA equivalents/g), and BA.2 (9.2 log_10_ RNA equivalents/g) virus groups compared to D614G (10.9 log_10_ RNA equivalents/g), Gamma (11.1 log_10_ RNA equivalents/g) and Delta (11.0 log_10_ RNA equivalents/g) viruses. VRNA was significantly lower in the BA.1 and BA.2 groups relative to the D614G, Gamma, and Delta virus groups (*p* = 0.0306, 0.0073, and 0.0014 and 0.0016, 0.0003, 0.0014 relative to BA.1 and BA.2, respectively). Mean infectious titers of BA.1 and BA.2 viruses in the lung were lower than the other viruses (3.8 log_10_ TCID_50_/g and 3.5 log_10_ TCID_50_/g, respectively, versus 6.6 log_10_ TCID_50_/g (D614G), 5.7 log_10_ TCID_50_/g (Alpha), 6.5 log_10_ TCID_50_/g (Gamma), 6.9 log_10_ TCID_50_/g (Delta), 4.9 log_10_ TCID_50_/g (BA.2)) (*p* < 0.0001). Further, the Alpha virus infectious titers were significantly lower than the Delta virus (*p* = 0.0073).

To investigate the cytokine responses of hamsters to SARS-CoV-2 VOC infection, we measured cytokine (*IL-1, IL-4, TNFα, IL-10, IL-2, IL-6*) and IFN response-related (*IFNγ, IFNλ, IRF1, iNOS*) gene expression by RT-qPCR at 3dpi in nasal turbinate (Fig. 3E) and lung (Fig. 3F) tissues (multiplexed protein-based assays to evaluate hamster analytes were unavailable). We observed a differential pattern of expression in response to infection in the upper (nasal turbinates) vs lower airway (lung). In contrast with our observations in BA.2-infected HNECs (Fig. 2C), we did not observe a significant increase of *IFNλ* in response to infection with any of the VOCs with the caveat that cytokine transcription levels may not correlate well with cytokine protein expression. Gene expression of pro-inflammatory cytokines *IL-1β, IL-2, IL-4*, and *IL-6*, as well as *IFNγ* was significantly upregulated in nasal turbinates in response to infection with all VOCs, compared to mock-infected hamster tissues (Fig. 3E). Pro-inflammatory gene expression was not significantly increased in the lungs of infected hamsters at 3 dpi (Fig. 3F). Levels of the interferon regulatory factor *IRF1* were strongly upregulated in the nasal turbinates and lungs in response to all VOCs except for BA.2, which could be due to lower replication of BA.2 in the hamster model. While expression of the anti-inflammatory cytokine *IL-10* was reduced in the nasal turbinates of hamsters infected with all VOCs, we observed a significant increase in the lungs, with the highest increases observed in response to Gamma and Alpha infection (Fig. 3E versus Fig. 3F). These results suggest that in this model of SARS-CoV-2 infection (1 × 10^4^ TCID_50_ delivered primarily to the upper respiratory tract), a strong pro-inflammatory cytokine response is induced in the upper airway, with little to no activation of pro-inflammatory innate responses in the lower airway (with anti-inflammatory *IL-10* expression) despite high levels of viral replication detected in the lungs.

**Fig. 4:**
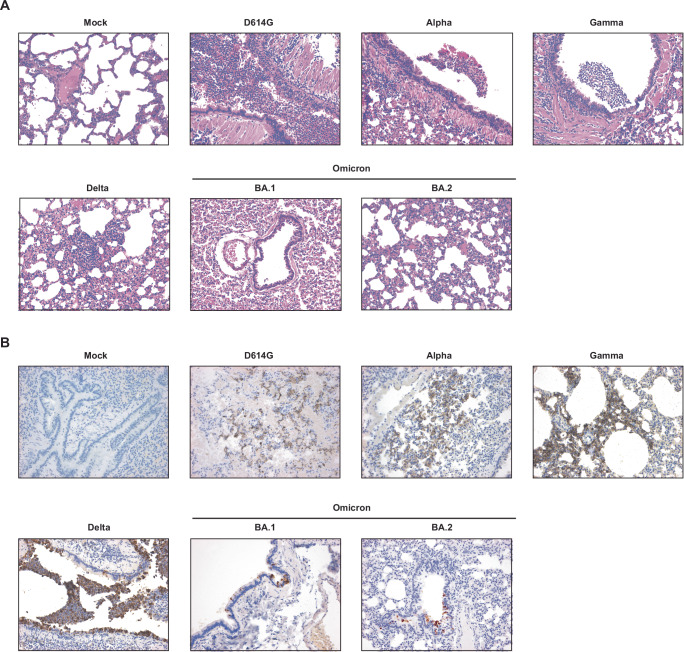
Histological analysis of lungs at 3 days after SARS-CoV-2 infection of golden hamsters. **A**, **B** Six-to-twelve-week-old mixed female and male golden hamsters (*Mesocricetus auratus*) were inoculated with 10^4^ TCID_50_ of SARS-CoV-2 or carrier only (control) by an intranasal route of administration in a volume of 20 μL. Representative hematoxylin and eosin (**A**) and immunohistochemistry (IHC, **B**) photomicrographs of the lungs for each experimental group are shown. Original magnification, 20x for each figure.

Histological analysis of the hamster lungs at 3 dpi was carried out (Fig. 4). Microscopic lesions in the nasal turbinates were minimal throughout, and consisted mainly of subepithelial accumulation of inflammatory cells, including scattered neutrophils (often marginating in vessels) and rare mononuclear cells with some edema. Occasionally, the inflammation extended to the epithelium, causing attenuation and release of neutrophils and cellular sloughing in the lumen. Pathology scores, which included subepithelial inflammation, epithelial damage, and exudate on the luminal aspect of the tissue, were not significantly different between inoculation groups (data not shown). Lung histopathology at 3 dpi (Fig. 4A) revealed inflammation predominately centered on the larger airways (i.e., bronchitis, tracheitis), which in some hamsters reached into the terminal bronchioles and alveoli. The exudate was mainly composed of neutrophils, cell debris, sloughed cells, and activated foamy macrophages (in alveoli). Mononuclear inflammation was present in the interstitium surrounding affected airways and adjacent vessels. Severe vascular changes were observed almost exclusively in the Gamma-inoculated group, being characterized by accumulation of mononuclear cells immediately subjacent the endothelium (endothelialitis). Lesion grading (Supplementary Fig. S1) showed that BA.1- and BA.2-infected hamsters had significantly lower average pathology scores compared to all the other groups (Kruskall–Wallis test with Dunn’s correction for multiple comparisons; highest, *p* = 0.015), except for the Alpha and mock-infected animals (lowest, *p* = 0.028). The Gamma-infected group had the highest pathology scores, albeit only significantly so compared to BA.1 and 2, and mock-infected animals (*p* = 0.0017 for all).

Immunohistochemistry for SARS-CoV-2 N protein in the lungs of infected hamsters was mainly finely granular and cytoplasmic, although extracellular labeling could be observed in areas of tissue damage (Fig. 4B). Reactivity was observed multifocally in the larger airways and in the alveoli, where it was mainly confined on the epithelial surface. Immunoreactivity did not exceed ~25% of the tissue area on section, with most cases (95%) having reactivity below 15%. Scoring of immunohistochemical reactivity showed that the mock- and BA.2-infected groups had significantly lower average IHC scored compared to all the other groups (Kruskall–Wallis test with Dunn’s correction for multiple comparisons; highest, *p* = 0.0285), except for Alpha- and BA.1-infected groups (lowest, *p* = 0.0726).

### SARS-CoV-2 transmission via directional airflow in golden hamsters

**Fig. 5:**
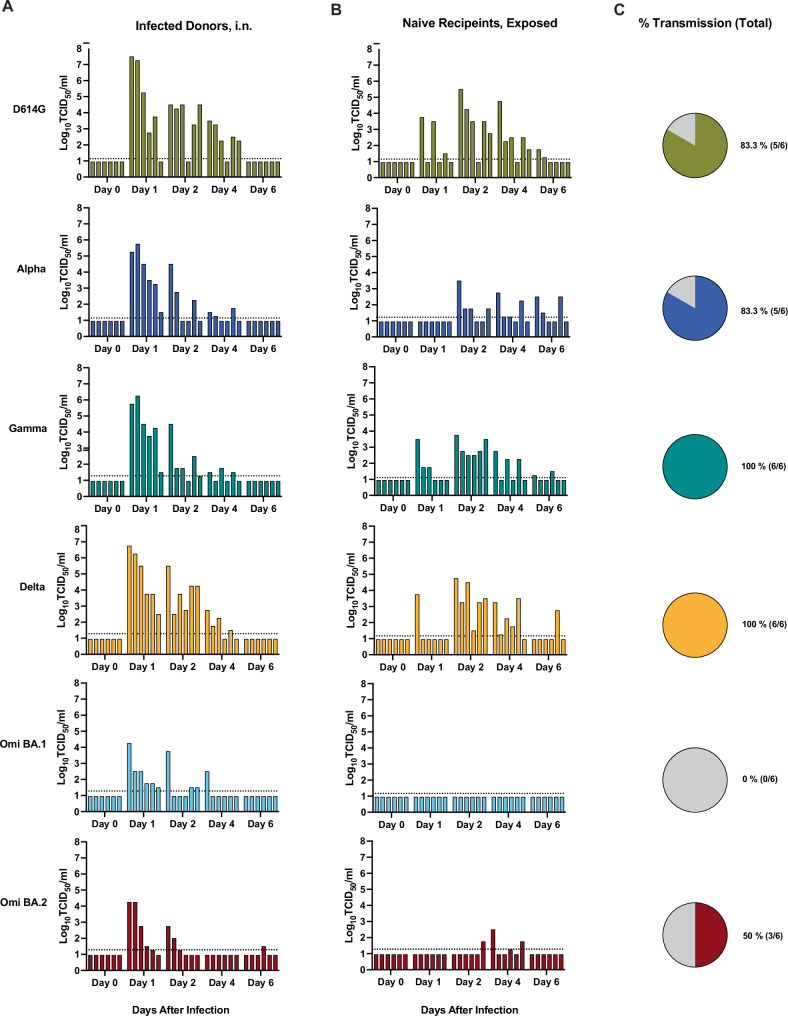
SARS-CoV-2 transmission by directional airflow in golden hamsters Six-to-twelve-week-old female (*n* = 3) and male (*n* = 3) Syrian golden hamsters (*Mesocricetus auratus*) were inoculated with 10^4^ TCID_50_ of each SARS-CoV-2 variant, D614G (gold), Alpha (dark blue), Gamma (teal), Delta (orange), Omicron BA.1 (light blue), and Omicron BA.2 (maroon) virus, by a low volume inoculum (20 µL) via the intranasal route of administration to serve as the donor animals. One day (24 h) after infection, donor animals were removed from the original cage and paired with a sex-matched naïve sentinel animal for co-housing in respiratory transmission unit with a separation of 9 cm between the two animals with directional airflow (air flow from donor to naïve sentinel). Oropharyngeal swab samples were obtained from donor (**A**) and recipient hamsters (**B**) on days 0, 1, 2, 4, and 6 after inoculation (**A**) or after directional airflow transmission unit housing exposure (**B**) and infectious titers at the indicated day after infection/exposure for each individual hamster are indicated by the bar heights. Bars in **A** and **B** are ordered to depict animal pairings in a discrete respiratory transmission unit. **C** The overall percent transmission is shown, indicating the number of naïve, exposed hamsters in each group that had at least one oropharyngeal swab above the limit of detection (LOD). The LOD is indicated by a dotted horizontal line. Three independent experiments were carried out, and the data were combined. (total *n* = 6).

Next we assessed the transmissibility of the SARS-CoV-2 variants by the respiratory route in golden hamsters in order to determine relative transmissibility in this animal model. Hamsters were inoculated intranasally (volume 20 μL) with 10^4^ TCID_50_ of respective SARS-CoV-2 variants (D614G, Alpha, Gamma, Delta, Omicron BA.1, or Omicron BA.2 viruses), and after 24 h were transferred to a respiratory transmission cage for co-housing with an age and sex-matched naïve hamster that was physically separated by a 9 cm air-permeable barrier under directional airflow (Supplementary Fig. S2). Oropharyngeal swab samples were obtained from inoculated donor (Fig. 5A) and naïve exposed hamsters (Fig. 5B) on days 0, 1, 2, 4, and 6 after viral inoculation or exposure, and transmission to a recipient hamster was deemed successful if at least one (of the 5) oropharyngeal swab yielded an infectious titer above the LOD. Infectious titers on the indicated day after inoculation/exposure for each individual hamster are indicated by the bar heights (Fig. 5A, B). Where statistics are reported, two-way ANOVA was carried out. After inoculation, all hamsters in each group were productively infected and had detectable virus except for one hamster from the BA.2 group. The mean infectious titers detected in the oropharyngeal swab samples from the directly inoculated hamsters peaked at 1 dpi (Fig. 5A and Supplementary Fig. S3). The mean infectious titers of D614G, Gamma, and Delta at 1 dpi were 6.9 log_10_ TCID_50_/ml, 5.6 log_10_ TCID_50_/ml, and 6.1 log_10_ TCID_50_/ml, respectively, and were greater than those of Alpha virus (5.1 log_10_ TCID_50_/ml), and significantly greater than BA.1 (3.5 log_10_ TCID_50_/ml) and BA.2 (3.8 log_10_ TCID_50_/ml) viruses (*p* = 0.0019, 0.0085, 0.0006 compared to BA.1, respectively, and *p* = 0.004, 0.0165, and 0.0013 compared to BA.2, respectively) (Supplementary Fig. 3). At 2 dpi, the mean infectious titers for BA.1 and BA.2 virus swabs were significantly lower than those of D614G and Delta virus (*p* = 0.0047 and 0.0016 for D614G and Delta compared to BA.1, respectively, and *p* = 0.0021 and 0.0007 for D614G and Delta compared to BA.2, respectively). Over the 6 days of the study, transmission occurred in more hamster pairings for D614G, Alpha, Gamma, and Delta viruses with 83.3%, 83.3%, 100%, and 100% transmission efficiency, respectively, compared to 0% and 50% for BA.1 and BA.2 viruses (Fig. 5C). Peak titres were lower for naïve, exposed recipients for all viruses, with transmission to at least one naïve individual by day 1 for D614G, Gamma and Delta viruses.

**Fig. 6:**
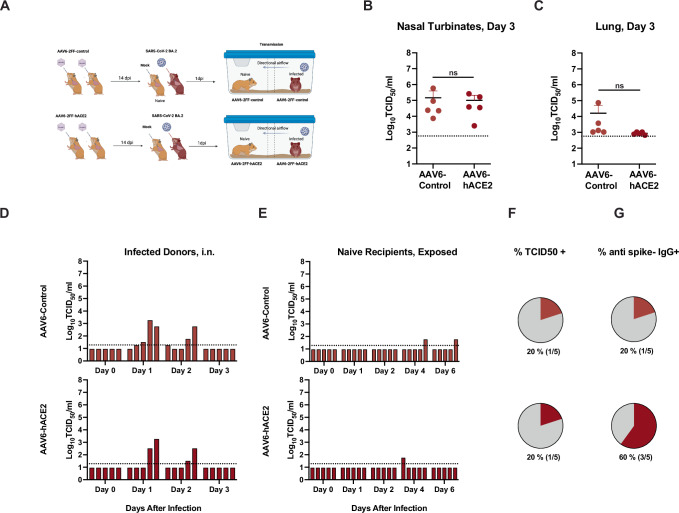
SARS-CoV-2 transmission by directional airflow in AAV6-hACE2 transduced golden hamsters. Six-to-twelve-week-old female (*n* = 3 donors and 3 recipients) and male (*n* = 2 donors and 2 recipients) Syrian golden hamsters (*Mesocricetus auratus*) were administered 10^11^ vector genomes (vg) of adeno-associated virus (AAV6.2FF) expressing hACE2 or a luciferase control by an intranasal route in a volume of 180 µL. After 14 days donor hamsters were inoculated with 10^4^ TCID_50_ of Omicron BA.2 (maroon) virus, by a low volume inoculum (20 µL) by the intranasal route of administration. One day (24 h) after infection, donor animals were removed from the original cage and paired with a sex-matched naïve sentinel animal for co-housing in a directional airflow transmission unit with a separation of 9 cm between the two animals (air flow from donor to naïve sentinel). **A** Schematic depiction of the experimental work flow. Infected donor animals were necropsied on day 3 after infection, and infectious titers detected in the nasal turbinates (**B**) and lungs (**C**) are shown. Unpaired *t* test, n.s. = not significant. The limit of detection (LOD) is indicated by a dotted line. Oropharyngeal swab samples were obtained from donor (**D**) and recipient hamsters (**E**) on days 0, 1, 2, 3 after inoculation (**D**) or 0, 1, 2, 4, 6 after respiratory transmission unit housing exposure (**E**), and infectious titers at the indicated day after infection/exposure for each individual hamster are indicated by the bar heights. Bars in **D** and **E** are ordered to depict animal pairings in a discrete respiratory transmission unit. **F** The overall percent transmission is shown, indicating the number of naïve, exposed hamsters in each group that had at least one oropharyngeal swab above the limit of detected. **G** The percent of the naïve hamsters that were sero-positive for IgG antibodies against the SARS-CoV-2 spike protein are shown. The LOD is indicated by a dotted horizontal line. A single experiment was carried (total *n* = 5). Panel **A** was created in BioRender (https://BioRender.com/g01q595).

In an additional experiment, we aimed to evaluate whether human ACE2 (hACE2) delivered to the upper and lower respiratory tract of hamsters could increase the transmission efficiency of the SARS-CoV-2 BA.2 variant (Fig. 6) in the event that the hamster ACE2 was less effective for Omicron variants. AAV6-2FF-ACE2 and the corresponding luciferase control vector were generated as described previously^66^. First, adult female (*n* = 3 donors) and male (*n* = 2 donors) wildtype hamsters were each administered 1 × 10^11^ vector genomes (vg) of adeno-associated virus (AAV6.2FF) expressing hACE2 or a luciferase control by an intranasal route twice in a volume of 90 μl. After 14 days, donor hamsters were inoculated with 10^4^ TCID_50_ of BA.2, by a low volume (20 μL) intranasal route of administration (Fig. 6A). One day (24 h) after infection, inoculated animals were transferred to a transmission cage and paired with a sex-matched naïve exposed animal for co-housing in an airborne transmission unit with a separation of 9 cm between the two animals with directional airflow (air flow from donor to naïve sentinel). Delivery of AAV6-hACE2 did not significantly increase the infectious titers in the nasal turbinates (Fig. 6B) or lungs (Fig. 6C) at 3 dpi after direct intranasal infection with BA.2. Of the inoculated donor hamsters, 3/5 of the AAV6-control hamsters and 2/5 of the AAV6-hACE2-exposed hamsters had detectable infectious virus in oropharyngeal swabs (Fig. 6D). After exposure to the infected donor hamsters via the respiratory route, 1/5 of the AAV6-control hamsters and 1/5 of the AAV6-hACE2 -exposed hamsters had detectable infectious BA.2 virus in oropharyngeal swabs (Fig. 6E), equivalent to a transmission efficiency of 20% based on detection of a positive swab sample (Fig. 6F). In addition, anti-spike IgG levels were evaluated in the naïve/exposed hamsters, and while the single virus positive hamster in the AAV6-control group had seroconverted, 3/5 hamsters in the AAV6-hACE2 group seroconverted. Since the serology data implied an increase in transmission with delivery of hACE2 we suggest further studies may be warranted.

## Discussion

In this study, we directly compared D614G, Alpha, Gamma, Delta, Omicron BA.1, and BA.2 variants, and assessed their replicative ability, pathogenesis, and transmissibility. Delta, BA.1, and BA.2 were found to replicate more efficiently in HNECs and dominate HNEC competition assays relative to D614G, Alpha, and Gamma viruses. BA.1 and BA.2, however, were observed to replicate poorly in IPSC human lung organoids, with Delta virus reaching the highest titer (Fig. 1). Further, BA.2 infection resulted in significantly more secretion of IFN-λ1, IFN-λ2, IFN-λ3, IL-6, and IL-1RA in HNECs. (Fig. 2). There were no substantial differences in weight loss among golden hamsters from the different variant groups (Fig. 3). Consistent with previous reports in Syrian hamsters, BA.1 and BA.2 viruses replicated at lower titers in the nasal turbinates and the lung tissues, compared to the other variants examined, and Gamma virus replicated to comparable titers to D614G and Delta viruses but caused greater lung pathology (Figs. 3, 4, and Supplemental Fig. 1). Lastly, the Gamma and Delta variants transmitted by the respiratory route with greater efficiency than D614G, Alpha, BA.1, and the Omicron BA.1 and BA.2 variants transmitted less efficiently by this mode than all the other viruses (Fig. 5). Lastly, efforts to overcome the deficiency in BA.2 respiratory transmission in hamsters via delivery of human ACE2 to the respiratory tract of donor and naïve hamsters were inconclusive (Fig. 6).

D614G had greater replicative fitness in Vero E6, Vero E6-TMPRSS2^OE^, and Calu-3 cell lines (Fig. 1). These data are aligned with previous studies that show that the spike P681R mutation, present in Delta virus, does not increase viral replication in Vero E6 or Vero E6-TMPRSS2^OE^ cell lines^67^. Delta virus is known to be highly efficient at using the TMPRSS2 entry pathway due to the P681R mutation and more efficient S1/S2 cleavage^68^. While it has been shown that BA.1 is less dependent on the TMPRSS2/plasma membrane fusion route for viral entry^69^, we noted that BA.1 and BA.2 reached a higher titer on 2 dpi in the presence of TMPRSS2 (Fig. 1A versus B, Vero E6 versus Vero E6-TMPRSS2^OE^). We also observed that Alpha virus titers were elevated compared to Delta and BA1 in the Calu-3 cell line (Fig. 1C), which is aligned with previously reported data^70^.

Differentiated HNECs have been shown to be susceptible to SARS-CoV-2 infection^22^, and infectious virus is released from the apical surface for a prolonged period despite the expression of antiviral cytokines^71^. We found that D614G, Alpha, and Gamma viruses had similar replication kinetics in HNECs, and that Delta, BA.1, and BA.2 viruses had similar titers that were significantly elevated compared to D614G, Alpha, and Gamma at 1 dpi, with Omicron BA.1 and BA.2 titers dropping at 3 dpi (Fig. 1D). Similar results were observed in a study that compared SARS-CoV-2, USA-WA1/2020 (WA1), Alpha, Beta, Delta, and Omicron viruses that showed Delta and Omicron virus titers being significantly higher compared to WA1 virus but dropping off at 4 dpi, while the other variants continued to increase in titer^70^. In contrast to a prior study that showed increased viral fitness for BA.2 virus compared to BA.1 virus in HNECs^29^, we observed that BA.1 and BA.2 viruses had comparable viral fitness (Fig. 1D). Results from other SARS-CoV-2 variant competition assays in HNECs^72^ were similar to our findings (Fig. 1F–N) with Delta virus out competing D614G virus, however contrary to our results, Gamma virus out competing D614G virus, D614G outcompeting Omicron virus, and Delta virus outcompeting Omicron virus. The results of our competition assays reflect the epidemiological patterns of emergence for Delta, BA.1 and BA.2, but not Alpha viruses. These differences may be due to experimental differences such as HNEC donor characteristics. Further, an important consideration in interpreting our competition assay data is the use of TCID_50_ to determine input ratios as the most relevant representation of viral infectiousness; genome copies may not accurately represent replication-competent viruses in a 1:1 ratio, and we are limited to genome copies for outputs since VOCs cannot be discerned by TCID_50_.

A published comparison of wildtype (WT), D614G, Alpha, and Beta as well as WT, Delta, and Omicron viruses (as separate experiments) in human primary lung tissues revealed a replicative advantage for D614G compared to Alpha virus^73^, that was congruent with our observations in IPSC-LOs (Fig. 1E). Further, we observed that Delta reached higher titers than Omicron BA.1 and BA.2 viruses in IPSC-LO s (Fig. 1E) which was similar to reported observations in human lung tissues, but differed from observations in human bronchial tissues, where Omicron achieved higher relative titers compared to Delta virus^73^. A recent study showed that Omicron and Alpha viruses reached lower titers in lung organoids compared to D614G, Beta, Gamma, and Kappa viruses^72^, which was similar to our observations; however, Delta virus reached the highest titers (Fig. 1E).

Several SARS-CoV-2 studies highlight the role of nasal epithelial immune responses as a key determinant of pathogenicity. In a published single-cell RNA sequencing study of nasopharyngeal swabs of mild and moderate COVID-19 cases revealed that despite similar viral loads, patients with mild symptoms showed strong induction of antiviral IFN response genes in the nose, whereas patients with more severe symptoms had relatively lower antiviral responses^74^. In a study comparing replication of Delta and Omicron in HNECs, Omicron significantly increased *IFNB* and *IFNL* expression compared to all other VOCs studied^70^. Here we show that notably BA.2 infection significantly increased the secretion of IFNλ1, IFNλ2, IFNλ3, IL-6, and IL-1RA compared to D614G in HNECs, a response not observed in IPSC-LOs. In IPSC-LOs, IL-8 and other inflammatory cytokines were upregulated across variants compared to the D614G lineage. IL-8 plays a key role in recruiting neutrophils and other immune cells to the site of infection; IL-8 and IL-6 detection together can be used as a predictor for disease severity in patients^75^. Additional studies have shown that early innate immune responses in the nasal epithelium have a direct impact on early viral replication levels, which has been correlated with the likelihood of transmission, and suggest that a robust early IFN response may contribute to milder disease outcomes associated with certain variants^76–78^. In addition, in vitro studies using HNECs have shown that these cells sustain a prolonged infection despite the expression of antiviral cytokines^71^. Similarly, in the hamster model, we observed different patterns of inflammatory gene expression in the upper versus lower airways. Gene expression of pro-inflammatory cytokines *IL-1β, IL-2, IL-4*, and *IL-6*, as well as *IFNγ* was significantly upregulated in nasal turbinates in response to infection with all VOCs, with the Gamma variant inducing the highest inflammatory response. There is a reciprocal expression of the anti-inflammatory cytokine *IL-10*, which is upregulated in the lungs but downregulated in the nasal turbinates. *IRF1*, a key regulator of the innate immune response and type III IFN production, was also upregulated in the lungs of hamsters^79^ although we did not observe a significant upregulation of *IFNλ*. Other studies have also shown a strong pro-inflammatory immune response in hamsters to SARS-CoV2 with a dysregulated IFN expression peaking between days 2 and 5^80,81^; Francis et al. showed higher inflammatory gene expression in the lungs compared to nasal turbinates^80^, while another comparative VOC study in hamsters observed elevated *IFNλ* expression 2 days post infection with ancestral, Alpha, Beta, and Gamma viruses^81^. A prior study has shown that the initial site of exposure to SARS-CoV-2 has an impact on the initial cytokine profile and downstream immune response in the lungs, with smaller volume inoculum localized in the nasal turbinates resulting in a less robust inflammatory response and reduced pathology compared to a higher volume inoculum delivered to the nasal turbinates and lungs^59^.

Golden (Syrian) hamsters are a well-established model for SARS-CoV-2 infection^82,83^, and have largely reflected disease severity associated with a particular VOC in humans. In upper and lower respiratory tract tissues of SARS-CoV-2-infected hamsters, we observed significantly lower infectious virus titers for the BA.1 and BA.2 viruses (Fig. 3A–D). While we observed lower Alpha virus mean titers relative to D614G in oropharyngeal swabs, nasal turbinates, and the lung (Fig. 3B–D), these differences were not significant and therefore align with previous work that showed comparable viral fitness of Alpha and D614G viruses in hamsters^40,84^. In addition, it has been shown that BA.1 variants are less pathogenic than the other circulating VOCs in both mice and hamsters^46,85–87^, which is consistent with clinical observations in humans that Omicron virus is associated with milder clinical severity^88^, although the widespread SARS-CoV-2 immunity from vaccination or natural infection has been shown to contribute in part to this phenomenon^89^. Hypotheses have been suggested to explain reduced Omicron replication in the lung, such as increased spike binding to hACE2 with reduced fusogenicity, reduced formation of syncytia and preference for the endosomal route for cellular entry^90^.

As expected, we noted modest pathology using a 20 µL SARS-CoV-2 administration volume and noted similar differential lung pathology severity for the different groups, with Gamma and D614G virus-infected hamsters presenting with greater lung pathology compared to Alpha and Delta virus-infected hamsters, and virtually no lung pathology in Omicron-infected hamsters (Fig. 4 and Supplemental Fig. 1). Increased lung pathology in hamsters infected with Gamma versus Alpha and ancestral virus has been reported^81^.

SARS-CoV-2 direct contact transmission between co-housed hamsters has been found to be very efficient and, therefore, unsuitable to discern transmission differences between variants. Even prior-exposed hamsters subjected to a homologous re-challenge four months following the initial infection are capable of transmitting SARS-CoV-2 when co-housed despite an absence of detected infectious virus^91^. Additional factors have been shown to be critical for transmission in this model, including the timing of exposure for directional airflow to coincide with peak infectious viral shedding of donor animals, typically at 1 dpi, which is the time we chose to expose the naïve to inoculated donor hamsters^92^. We opted to use the D614G virus as the comparator group since in previous studies, D614G has been shown to have a higher replicative fitness^21,22,93^ and transmisiblity^21,22,40^ advantage in hamsters compared to ancestral SARS-CoV-2, which matches what was observed clinically in humans^13,14^. Moreover, the D614G substitution is shared by all the variants included in this work.

Our data showed that Gamma and Delta viruses transmitted most efficiently, followed by D614G and Alpha viruses, and with much lower transmission with BA.2 and no transmission with BA.1 (Fig. 5). These data are aligned with previously published data showing that Alpha virus and D614G had similar transmissibility in hamsters^40,84^. These data do not align with the spread of Alpha virus in humans; however, this could be a limitation of the directional airflow transmission setup that we used (9 cm). A published transmission study that used a larger separation distance thereby restricting SARS-CoV-2 transmission to smaller particles below <5 μm showed a transmission fitness advantage for Alpha virus relative to ancestral virus^43^. Delta virus transmission in hamsters has been shown to be comparable to Alpha virus with a 16.5 cm separation between animals^44^ and to have increased competitiveness relative to an ancestral isolate in competitive respiratory transmission experiments^47^. SARS-CoV-2 Omicron lineages have shown variable capacity to replicate and transmit in golden hamsters. While one study reported enhanced respiratory transmission efficiency of Omicron BA.1 virus relative to Delta virus in hamsters^46^, most studies have shown none or significantly reduced transmission potential of Omicron BA.1^42,48^ and BA.2 in hamsters^42,49^. These studies align with our observations of greatly reduced transmission by the respiratory route for BA.1 and BA.2 (Fig. 5). Subsequent Omicron viruses such as the XBB1.5 lineage viruses have shown much greater respiratory transmissibility in hamsters compared to BA.1 and BA.2^49^.

Transgenic hACE2 hamsters are more susceptible to infection with SARS-CoV-2 isolate USA-WA1/2020 strain^94^ as well as BA.1 and BA.2 lineages^95^. Similarly, transgenic hamsters that express human ACE2 under the human keratin-18 (K18) promoter succumb to SARS-CoV-2 infection^94,96^. The lack of mortality may be due to our use of the cytomegalovirus (CMV) promoter to drive hACE2 expression, instead of the K18 promoter, in the above studies resulting in lethal infection in mice^97^, and associated with neurodissemination^98^.

Additional investigation will be required to determine the mechanism(s) responsible for the increased fitness and transmissibility of a particular variant in the hamster model of infection, such as ACE2 binding and mechanism of virus entry, genome synthesis, viral budding/cell-to-cell spread, modulation of the innate immune response, escape from the adaptive response, and transmissibility in partially immune hosts. Increasing our understanding of these models of SARS-CoV-2 infection and standardizing this approach will further enhance our ability to assess and respond to future VOCs, evaluate differences in viral pathogenicity and transmission, and evaluate vaccines and therapeutics.

We note several limitations to the work reported in this study. While statistically notable differences were observed between VOCs in a number of experiments, greater distinction of VOC phenotypes may have been discerned, had additional data points been added, particularly for ex vivo and transmission studies, though the number of viruses evaluated in tandem would have been more limited. Despite the propagation of virus stocks in VeroE6- based cell lines, additional ex vivo experiments with inactivated virus stock followed by infection could identify cytotoxic cytokine or other pro-inflammatory activity that may modulate infection. While golden hamsters are a well-established model for SARS-CoV-2 pathogenesis and transmission, the dose and volume that we used to inoculate the donor animals in this study results in only mild-to-moderate infection; therefore, this work does not model severe COVID-19 in humans. Also, these studies were carried out in immunologically naïve animals. This is an advantage when comparing the inherent transmissibility, but does not model current human infections since the majority of people have been vaccinated against the spike protein and/or previously infected with SARS-CoV-2. Lastly, it is unclear if the attenuation of Omicron isolate replication in the lung that is observed in hamsters also occurs in humans.

## Supplementary information

Supplementary Information

## Data Availability

The datasets used and/or analysed during the current study are available from the corresponding author, Dr. Samira Mubareka, Sunnybrook Research Institute (samira.mubareka@sunnybrook.ca), on reasonable request. This paper does not report original code. Any additional information required to re-analyze the data described in this paper is available upon request from the lead contact.
