# Supplementary material for: Pathogenesis and transmission of SARS-CoV-2 D614G, Alpha, Gamma, Delta, and Omicron variants in golden hamsters
Source: Npj Viruses. 2025 Feb 24;3:15. doi: 10.1038/s44298-025-00092-2 (PMC11850601; doi:10.1038/s44298-025-00092-2)
Supplement: Supplementary file 1 — Supplementary Information [file 44298_2025_92_MOESM1_ESM.pdf]

Supplementary Material

Pathogenesis and Transmission of SARS-CoV-2 D614G, Alpha, Gamma, Delta, Omicron variants in golden hamsters

Andra Banete[#,1], Bryan D Griffin[#,1], Juan C Corredor[1], Emily Chien[1], Lily Yip[1], Tarini N A Gunawardena[2,3], Kuganya Nirmalarajah[1], Jady Liang[4,5] , Yaejin Lee[1], Alexander Leacy[6], Sara Pagliarani[6], Richard de Borja[7], Winfield Yim[1], Hunsang Lee[8], Yu Onodera[4,9], Patryk Aftanas[10], Patrick Budylowski[11,12,17], Sang Kyun Ahn[13], Yanlong Pei[6], Hong Ouyang[3], Laura Kent[14], Xinliu Angel Li[15], Mario A Ostrowski[11,16,17], Robert A Kozak[1,10,18], Sarah K. Wootton[6], Natasha Christie-Holmes[19], Scott D Gray-Owen[13,19], Mikko Taipale[8,13], Jared T. Simpson[13,7,20], Finlay Maguire[10,21,22], Allison J McGeer[23, 17], Haibo Zhang[4,5,24,25], Leonardo Susta[6], Theo J Moraes[3,26], Samira Mubareka[1,18*]

Affiliations

[1]Biological Sciences, Sunnybrook Research Institute, Toronto, ON M4N 3M5, Canada.
[2]Programme in Molecular Medicine, The Hospital for Sick Children, Toronto, ON M5G 0A4, Canada.
[3]Program in Translational Medicine, Hospital for Sick Children, Toronto, ON M5G 1X8, Canada.
[4]Keenan Research Centre for Biomedical Science, St. Michael's Hospital, Unity Health Toronto, Toronto, ON M5B 1W8, Canada.
[5]Department of Physiology, University of Toronto, Toronto, ON M5S 1A8, Canada.
[6]Department of Pathobiology, University of Guelph, Guelph, ON N1G 2W1, Canada .
[7]Ontario Institute for Cancer Research, Toronto, ON M5G 0A3, Canada.
[8]Donnelly Centre for Cellular and Biomolecular Research, University of Toronto, Toronto, ON M5S M5S 3E1, Canada.
[9]Department of Emergency and Critical Care Medicine, Faculty of Medicine, Yamagata University, Yamagata 990-9585, Japan.
[10]Shared Hospital Laboratory, Toronto, ON M4N 3M5, Canada .
[11]Department of Medicine, University of Toronto, Toronto, ON M5S 1A8, Canada.
[12]Institute of Medical Science, University of Toronto, Toronto, ON M5S 1A8,, Canada.
[13]Department of Molecular Genetics, University of Toronto, Toronto, ON M5S 1A8, Canada.
[14]Division of Comparative Medicine, Faculty of Medicine, University of Toronto, Toronto, Ontario, Canada.
[15]Department of Microbiology, Sinai Health System, Toronto, Ontario, Canada
[16]Department of Immunology, University of Toronto, Toronto, ON M5S 1A8, Canada.
[17]Keenan Research Centre for Biomedical Science of St. Michael's Hospital, Unity Health Toronto, Toronto, ON M5B 1T8, Canada.
[18]Department of Laboratory Medicine and Pathology, University of Toronto, Toronto, ON, Canada.
[19]Toronto High Containment Facility, Temerty Faculty of Medicine, University of Toronto, Toronto, ON M5S3E1, Canada,.
[20]Department of Computer Science, University of Toronto, Toronto, ON M5S 2E4, Canada.
[21]Department of Community Health and Epidemiology, Faculty of Medicine, Dalhousie University, Halifax, NS B3H 4R2, Canada.
[22]Faculty of Computer Science, Dalhousie University, Halifax, NS B3H 4R2, Canada.

[23]Division of Infection Prevention and Control, Sinai Health System, University of Toronto, ON, Canada.
[24]Interdepartmental Division of Critical Care Medicine, University of Toronto, Toronto, ON M5G 2N2, Canada.
[25]Department of Anaesthesiology and Pain Medicine, University of Toronto, Toronto, ON M5G 1E2, Canada.
[26]Division of Respiratory Medicine, Department of Pediatrics, Hospital for Sick Children, Toronto, ON M5G 1X8, Canada.

*Corresponding author: Dr. Samira Mubareka (samira.mubareka@sunnybrook.ca)

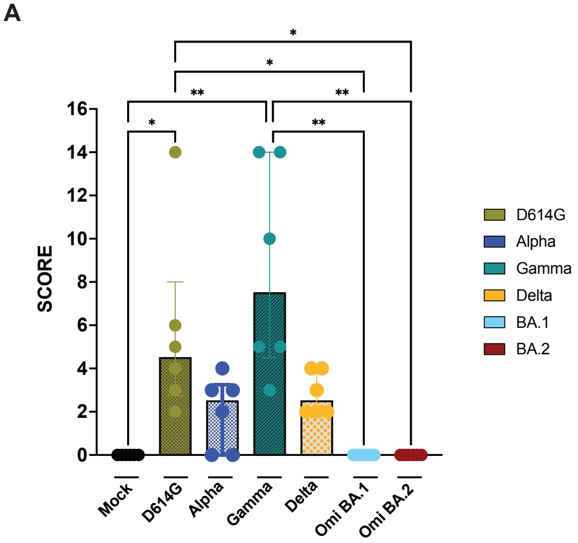

Figure S1: Histology scores of SARS-CoV-2-infected hamster lungs.
Six-to-twelve-week-old female and male golden hamsters (Mesocricetus auratus) were mock inoculated (black) or inoculated with 10^4 $TCID_{50}$ of each SARS-CoV-2 variant, D614G (gold), Alpha (dark blue), Gamma (teal), Delta (orange), Omicron BA.1 (light blue), and Omicron BA.2 (maroon) virus, using a low volume inoculum (20 uL) via the intranasal route of administration. Bar height indicates the median histology scores and error bars indicate interquartile range. Average histopathological scores were compared by the non-parametric Kruskall-Wallis test with Dunn's test for multiple pairwise comparisons. Significant between groups are indicated by binary connectors: *p $\leq$0.05, **p$\leq$0.01 ***.

A

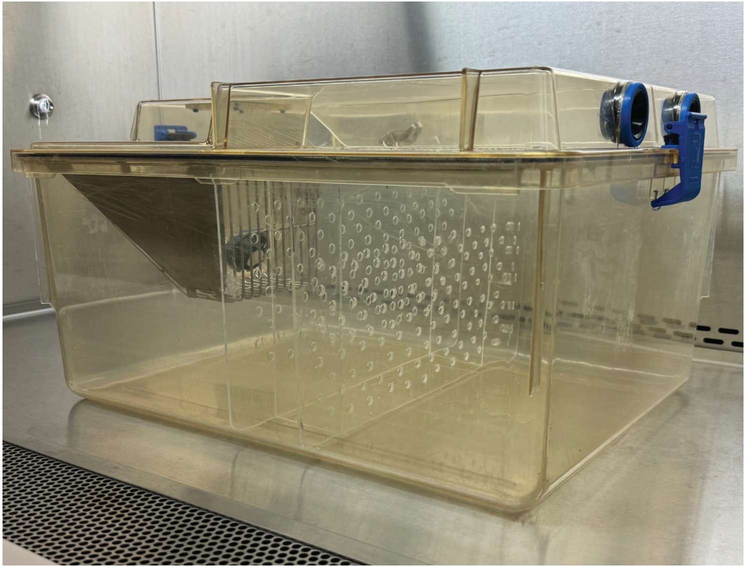

Figure S2: Airborne transmission unit for directional airflow transmission experiments.
Photo depiction of a prototypical hamster isolation cage used for the transmission studies.

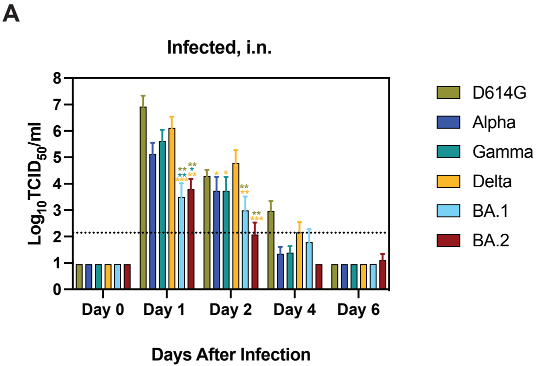

Figure S3: SARS-CoV-2 transmission experiment mean shedding by variant.
Six-to-twelve-week-old female (n = 3) and male (n = 3) Syrian golden hamsters (Mesocricetus auratus) were inoculated with 104 TCID50 of each SARS-CoV-2 variant, D614G (gold), Alpha (dark blue), Gamma (teal), Delta (orange), Omicron BA.1 (light blue), and Omicron BA.2 (maroon) virus, by a low volume inoculum (20 uL) via the intranasal route of administration to serve as the donor animals. One day (24 hours) after infection, donor animals were removed from the original cage and paired with a sex-matched naïve sentinel animal for co-housing in an directional airflow transmission unit with a separation of 9 cm between the two animals with directional airflow (air flow from donor to naïve sentinel). A) Mean oropharyngeal swab samples were obtained from donor 0, 1, 2, 4, and 6 after inoculation and infectious titers at the indicated day after infection/exposure for each individual hamster are indicated by the bar heights. The LOD is indicated by a dotted horizontal line. Three independent experiments were carried out and the data were combined (total n = 6).

Supplementary Table 1: Primer/probe sequences

Target	Oligo	Sequence (5'→3')
SARS-CoV-2 E gene	Forward	ACAGGTACGTTAATAGTTAATAGCGT;
	Reverse	ATATTGCAGCAGTACGCACACA
	Probe	ACACTAGCCATCCTTACTGCGCTTCG
IL-1β	Forward	GGCTGATGCTCCCATTCG
	Reverse	CACGAGGCATTTCTGTTGTTCA
	Probe	CAGCTGCACTGCAGGCTCCGAG
IL-6	Forward	CCTGAAAGCACTTGAAGAATTCC
	Reverse	GGTATGCTAAGGCACAGCACACT
	Probe	AGAAGTCACCATGAGGTCTACTCGGCAAAA
TNF-α	Forward	GGAGTGGCTGAGCCATCGT
	Reverse	AGCTGGTTGTCTTTGAGAGACATG
	Probe	CCAATGCCCTCCTGGCCAACG
IL-2	Forward	GTGCACCCACTTCAAGCTCTAA
	Reverse	AAGCTCCTGTAAGTCCAGCAGTAAC
	Probe	AGGAAACCCAGCAGCACCTCGAGC
IL-4	Forward	CCACGGAGAAAGACCTCATCTG
	Reverse	GGGTCACCTCATGTTGGAAATAAA
	Probe	CAGGGCTTCCCAGGTGCTTCGCAAGT
IL-10	Forward	GTTGCCAAACCTTATCAGAAATGA
	Reverse	TTCTGGCCCGTGGTTCTCT
	Probe	CAGTTTTACCTGGTAGAAGTGATGCCCCAGG
IRF1	Forward	GGCATACAACATGTCTTCACG
	Reverse	GCTATGCTTTGCCATGTCAA
	Probe	CACAATGACGCCAGACCTTGCTCA
IFNλ	Forward	CCCACCAGATGCAAAGGA
	Reverse	CTTGAGCAGCCACTCTTCTAT
	Probe	ACATAGCCCGGTTCAAGTCTCTGC
IFNγ	Forward	GGCCATCCAGAGGAGCATAG
	Reverse	TTTCTCCATGCTGCTGTTGAA
	Probe	CACCATCAAGGCAGACCTGTTTGCTAACTT
RPL18	Forward	GTTTATGAGTCGCACTAACCG
	Reverse	TGTTCTCTCGGCCAGGAA
	Probe	TCTGTCCCTGTCCCGGATGATC

Supplementary Table 2: Raw dataset for Figure 2 cytokines, chemokines, and growth analyses.

	15-Plex															9-Plex								
**Sample name**	**GM-CSF**	**IFNγ**	**IL-1β**	**IL-1RA**	**IL-2**	**IL-4**	**IL-5**	**IL-6**	**IL-8**	**IL-10**	**IL-12p40**	**IL-12p70**	**IL-13**	**MCP-1**	**TNFα**	**IFNα-2**	**IFNβ**	**IFNγ**	**IFNγR1**	**IFNε**	**IFNλ1**	**IFNλ2**	**IFNλ3**	**IFNω**
HNE donor 1 D614G #1	31.45	1.09	1.34	85.84	0.42	0.10	0.05	32.40	9,352.32	0.26	2.44	OOR <	0.81	109.95	4.77	OOR <	OOR	OOR	27.34	OOR <	4.36	21.85	6.31	OOR <
HNE donor 1 D614G #2	67.82	1.16	2.18	33.84	0.77	0.12	0.06	40.27	11,490.65	0.20	3.40	0.65	OOR <	282.63	7.67	OOR <	OOR	OOR	30.57	OOR <	4.00	22.92	5.58	OOR <
HNE donor 2 D614G #1	103.02	1.19	2.18	21.20	0.62	0.10	0.12	13.00	9,698.30	0.32	3.40	OOR <	0.03	66.72	6.41	OOR <	OOR	OOR	27.34	OOR <	5.05	8.22	5.29	OOR <
HNE donor 2 D614G #2	61.18	1.14	0.39	26.36	0.38	0.13	0.08	9.05	9,807.94	0.20	5.25	0.38	OOR <	39.68	4.69	OOR <	OOR	OOR	32.21	OOR <	7.16	8.22	8.28	OOR <
HNE donor 3 D614G #1	41.85	1.09	2.18	3.10	0.38	0.12	0.08	5.65	10,576.35	0.37	8.77	0.38	OOR <	187.35	6.93	OOR <	OOR	OOR	25.27	OOR <	1.59	8.22	3.66	OOR <
HNE donor 3 D614G #2	69.00	1.15	0.73	2.08	0.30	0.11	0.06	5.22	10,474.10	0.18	5.25	0.38	2.60	267.21	7.01	OOR <	OOR	OOR	25.11	OOR <	1.59	8.22	3.66	OOR <
HNE donor 4 D614G #1	23.40	0.78	0.56	30.45	0.73	0.10	0.05	16.79	8,403.81	0.20	7.03	1.15	OOR <	188.83	5.67	OOR <	OOR	OOR	29.92	OOR <	4.27	37.07	3.66	OOR <
HNE donor 4 D614G #2	28.67	0.93	3.99	10.37	0.38	0.13	0.04	41.48	9,355.33	0.09	3.40	0.38	OOR <	1,369.65	12.87	OOR <	OOR	OOR	16.65	OOR <	1.59	9.58	3.66	OOR <
HNE donor 1 B117 #1	22.32	0.89	1.34	19.24	0.04	0.12	0.04	19.28	10,121.81	0.15	5.25	OOR <	OOR <	176.78	3.70	OOR <	OOR	OOR	20.74	OOR <	4.33	12.34	3.66	OOR <
HNE donor 1 B117 #2	57.03	1.19	1.04	44.19	0.21	0.11	0.05	46.59	9,976.24	0.32	0.35	0.38	1.44	323.38	8.92	OOR <	OOR	OOR	26.70	OOR <	12.88	34.28	11.57	OOR <
HNE donor 2 B117 #1	50.62	1.28	1.63	4.24	0.21	0.10	0.05	18.26	9,411.37	0.15	3.40	0.12	OOR <	72.95	5.67	OOR <	OOR	OOR	27.02	OOR <	5.08	8.22	3.66	OOR <
HNE donor 2 B117 #2	39.86	1.16	1.04	14.15	0.13	0.10	0.06	9.42	9,280.45	0.26	2.44	0.12	0.03	71.02	5.48	OOR <	OOR	0.05	23.53	OOR <	12.59	8.22	5.73	OOR <
HNE donor 3 B117 #1	65.86	1.16	1.04	2.80	OOR	0.10	0.05	10.11	9,077.30	0.15	OOR <	0.12	OOR <	217.58	7.12	OOR <	OOR	OOR	21.97	OOR <	1.59	8.22	3.66	OOR <
HNE donor 3 B117 #2	61.68	1.11	1.04	4.29	0.26	0.10	0.04	9.15	10,031.69	0.32	3.40	0.65	5.17	204.89	5.63	OOR <	OOR	OOR	25.11	OOR <	1.59	8.22	3.66	OOR <
HNE donor 4 B117 #1	16.26	0.86	1.34	442.86	0.54	0.10	0.07	16.94	9,219.78	0.23	3.40	0.90	OOR <	153.00	5.29	OOR <	OOR	OOR	32.70	OOR <	10.49	35.27	4.25	OOR <
HNE donor 4 B117 #2	19.95	1.08	1.63	226.56	0.21	0.12	0.07	27.52	8,941.15	0.12	3.40	0.65	0.81	111.15	6.27	OOR <	OOR	0.01	29.27	OOR <	16.60	49.83	9.08	OOR <
HNE donor 1 P.1 #1	35.52	0.93	0.73	10.67	0.04	0.11	0.03	62.33	8,789.13	0.20	5.25	OOR <	4.18	262.60	6.12	OOR <	OOR	OOR	23.53	OOR <	3.90	21.15	4.55	OOR <
HNE donor 1 P.1 #2	25.27	1.01	1.34	32.47	0.13	0.12	0.04	27.87	8,176.64	0.20	4.33	0.12	1.44	156.80	4.39	OOR <	OOR	0.07	33.52	OOR <	2.84	14.32	4.55	OOR <
HNE donor 2 P.1 #1	38.07	0.96	1.63	5.18	0.30	0.10	0.07	17.12	8,861.85	OOR <	5.25	0.38	OOR <	82.71	3.63	OOR <	OOR	OOR	26.86	OOR <	2.72	8.22	3.66	OOR <
HNE donor 2 P.1 #2	49.08	0.96	0.73	9.34	0.17	0.09	0.06	9.34	9,457.21	0.09	OOR <	OOR <	0.03	33.44	3.24	OOR <	OOR	OOR	31.88	OOR <	2.50	8.22	3.66	OOR <
HNE donor 3 P.1 #1	42.20	0.80	1.04	4.71	OOR	0.07	0.05	3.33	8,606.84	0.09	1.44	0.38	1.44	173.06	4.43	OOR <	OOR	OOR	25.43	OOR <	1.59	8.22	3.66	OOR <
HNE donor 3 P.1 #2	107.55	1.02	1.04	3.82	OOR	0.09	0.05	17.97	9,094.56	0.04	10.48	0.52	2.60	263.55	8.66	OOR <	OOR	OOR	27.34	OOR <	1.59	8.22	3.66	OOR <
HNE donor 4 P.1 #1	8.28	1.03	1.34	96.86	0.17	0.11	0.04	23.31	9,199.20	0.15	7.90	OOR <	0.03	174.78	5.48	OOR <	OOR	OOR	34.18	OOR <	5.63	26.18	3.66	OOR <
HNE donor 4 P.1 #2	10.81	0.85	1.63	20.16	0.30	0.09	0.00	19.49	8,092.32	0.15	2.44	OOR <	2.60	96.54	4.05	OOR <	OOR	OOR	23.53	OOR <	4.27	23.86	3.66	OOR <
HNE donor 1 Delta #1	31.45	0.82	1.04	22.40	0.04	0.13	0.06	48.42	8,675.81	0.26	3.40	0.38	OOR <	254.31	5.11	OOR <	OOR	OOR	22.75	OOR <	2.96	10.95	3.66	OOR <
HNE donor 1 Delta #2	22.32	1.16	0.73	15.71	0.04	0.08	0.02	45.31	8,362.11	0.15	6.14	0.25	0.81	303.58	5.14	OOR <	OOR	OOR	26.22	OOR <	1.59	8.22	3.66	OOR <
HNE donor 2 Delta #1	54.38	1.03	1.63	36.85	0.46	0.09	0.07	19.03	9,292.37	0.23	5.25	OOR <	OOR <	72.43	6.00	OOR <	OOR	OOR	30.57	OOR <	5.37	8.22	3.66	OOR <
HNE donor 2 Delta #2	38.12	1.02	1.34	16.69	OOR	0.11	0.05	17.28	7,427.92	0.15	6.14	OOR <	OOR <	36.40	4.77	OOR <	OOR	OOR	21.66	OOR <	11.57	8.22	8.82	OOR <
HNE donor 3 Delta #1	57.89	0.93	1.34	2.82	OOR	0.11	0.04	6.56	8,670.48	0.04	OOR <	0.38	OOR <	263.84	5.97	OOR <	OOR	OOR	26.06	OOR <	1.59	8.22	3.66	OOR <
HNE donor 3 Delta #2	49.08	0.93	1.04	8.70	OOR	0.07	0.05	5.58	8,406.35	0.04	4.33	OOR <	1.44	192.11	5.14	OOR <	OOR	OOR	24.16	OOR <	1.59	8.22	3.66	OOR <
HNE donor 4 Delta #1	17.69	0.96	1.77	212.79	0.30	0.10	0.03	10.24	8,236.50	0.26	5.25	OOR <	1.44	115.87	4.12	OOR <	OOR	OOR	31.88	OOR <	4.17	18.36	3.66	OOR <
HNE donor 4 Delta #2	17.49	0.80	3.99	70.68	0.13	0.11	0.04	37.10	7,953.27	0.09	7.03	0.38	2.60	635.18	14.98	OOR <	OOR	OOR	23.85	OOR <	3.00	25.65	3.66	OOR <
HNE donor 1 BA.1 #1	31.41	0.99	0.73	92.67	0.04	0.08	0.04	54.76	8,874.30	0.20	7.03	0.12	OOR <	185.17	5.11	OOR <	OOR	0.03	24.79	OOR <	11.64	30.80	11.45	OOR <
HNE donor 1 BA.1 #2	14.79	0.99	0.52	55.17	OOR	0.09	OOR <	55.23	8,253.73	0.26	1.44	OOR <	OOR <	226.27	4.54	OOR <	OOR	OOR	24.32	OOR <	11.21	27.44	9.34	OOR <
HNE donor 2 BA.1 #1	37.60	1.01	2.71	22.59	0.21	0.07	0.09	40.33	9,146.64	0.07	1.44	0.12	OOR <	70.08	10.88	OOR <	OOR	OOR	30.90	OOR <	68.82	19.48	40.22	OOR <
HNE donor 2 BA.1 #2	39.66	1.02	1.04	12.32	OOR	0.06	0.05	33.95	8,960.80	0.26	0.35	OOR <	OOR <	64.18	12.00	OOR <	OOR	OOR	41.82	OOR <	82.79	24.23	47.92	OOR <
HNE donor 3 BA.1 #1	46.89	1.08	1.04	4.86	OOR	0.11	0.04	11.20	8,449.68	0.15	3.40	0.38	OOR <	205.89	6.15	OOR <	OOR	OOR	27.82	OOR <	4.81	10.19	3.66	OOR <
HNE donor 3 BA.1 #2	61.89	1.11	0.73	2.95	0.28	0.09	0.05	11.69	7,646.79	OOR <	1.44	OOR <	1.44	231.72	8.48	OOR <	OOR	OOR	27.34	OOR <	3.61	8.22	3.66	OOR <
HNE donor 4 BA.1 #1	42.39	1.05	6.59	55.00	0.13	0.10	0.02	166.46	7,911.60	0.07	7.03	OOR <	1.44	1,340.75	37.16	OOR <	OOR	OOR	28.63	OOR <	3.90	22.34	3.66	OOR <
HNE donor 4 BA.1 #2	10.06	0.80	1.63	63.71	0.30	0.11	0.02	16.51	7,923.14	0.09	OOR <	OOR <	OOR <	124.58	4.31	OOR <	OOR	OOR	33.19	OOR <	5.11	16.95	3.66	OOR <
HNE donor 1 BA.2 #1	20.27	1.06	1.34	335.46	0.13	0.12	0.02	71.89	8,331.98	0.18	5.25	OOR <	0.03	138.81	15.30	OOR <	OOR	OOR	27.82	OOR <	57.27	106.93	51.65	OOR <
HNE donor 1 BA.2 #2	13.57	0.79	1.34	239.32	0.26	0.07	0.05	30.48	9,167.02	OOR <	7.03	OOR <	OOR <	74.57	15.73	OOR <	OOR	OOR	26.86	OOR <	49.12	104.10	45.52	OOR <
HNE donor 2 BA.2 #1	22.90	1.34	1.63	214.44	0.77	0.12	0.08	223.21	9,834.04	0.04	7.03	OOR <	OOR <	248.47	55.30	OOR <	OOR	OOR	42.15	OOR <	413.81	229.93	257.05	OOR <
HNE donor 2 BA.2 #2	42.15	1.27	2.45	14.69	0.13	0.11	0.06	64.40	8,957.99	0.09	3.40	OOR <	0.03	603.20	10.96	OOR <	OOR	OOR	35.01	OOR <	75.31	150.93	38.91	OOR <
HNE donor 3 BA.2 #1	29.12	1.24	0.89	23.44	0.46	0.10	0.06	65.66	9,417.46	0.09	7.03	0.65	1.44	525.97	10.70	OOR <	OOR	OOR	36.17	OOR <	95.05	175.69	46.98	OOR <
HNE donor 3 BA.2 #2	39.95	1.32	1.04	26.58	0.21	0.15	0.03	91.71	10,391.32	0.15	6.14	0.38	2.60	695.64	10.34	OOR <	OOR	OOR	40.82	OOR <	91.82	183.09	41.73	OOR <
HNE donor 4 BA.2 #1	5.82	0.92	1.34	248.52	0.21	0.09	0.03	23.62	8,113.87	0.09	5.25	0.12	OOR <	120.54	14.66	OOR <	OOR	OOR	27.98	OOR <	69.09	171.77	37.45	OOR <

Supplementary Table 3: Raw dataset for Figure 2 cytokines, chemokines, and growth analyses.

	15-Plex															9-Plex								
Sample name	GM-CSF	IFNγ	IL-1β	IL-1RA	IL-2	IL-4	IL-5	IL-6	IL-8	IL-10	IL-12p40	IL-12p70	IL-13	MCP-1	TNFα	IFNα-2	IFNβ	IFNγ	IFNγR1	IFNε	IFNλ1	IFNλ2	IFNλ3	IFNω
LBO mock #1	OOR <	0.30	1.91	0.33	0.46	0.13	0.04	8.90	285.71	0.52	8.77	0.77	0.81	329.49	1.28	OOR <	OOR	OOR	7.07	OOR <	OOR <	OOR <	1.00	OOR <
LBO mock #2	0.12	0.27	2.18	0.33	0.46	0.13	0.06	12.04	400.42	0.72	1.44	0.52	OOR <	338.51	1.04	OOR <	OOR	OOR	9.03	OOR <	OOR <	OOR <	0.43	OOR <
LBO mock #3	OOR <	0.26	1.48	0.33	0.30	0.13	0.04	1.05	156.71	0.52	6.14	OOR <	3.66	174.05	0.71	OOR <	OOR	0.01	5.96	OOR <	OOR <	OOR <	0.43	OOR <
LBO mock #4	OOR <	0.26	1.63	0.33	0.54	0.13	0.09	5.31	341.73	0.55	7.03	OOR <	OOR <	202.38	0.79	OOR <	OOR	0.09	6.24	OOR <	OOR <	OOR <	1.59	OOR <
LBO mock #5	OOR <	0.34	1.77	0.43	0.62	0.13	0.05	2.65	375.81	0.52	5.25	0.90	1.44	145.62	0.88	OOR <	OOR	OOR	6.24	OOR <	OOR <	OOR <	0.72	OOR <
LBO mock #6	0.00	0.26	1.34	0.38	0.26	0.14	0.04	28.95	180.38	0.52	5.25	0.52	1.44	252.55	1.52	OOR <	OOR	OOR	10.43	OOR <	OOR <	OOR <	1.29	OOR <
LBO D614G#1	0.45	0.26	1.48	0.33	0.09	0.13	0.05	4.36	222.43	0.52	10.48	0.65	OOR <	199.87	1.28	OOR <	OOR	OOR	6.79	OOR <	OOR <	OOR <	1.29	OOR <
LBO D614G#2	0.45	0.26	1.34	0.33	0.73	0.13	0.05	3.31	207.67	0.52	5.25	0.52	OOR <	164.09	1.28	OOR <	OOR	OOR	6.93	OOR <	OOR <	OOR <	1.59	OOR <
LBO D614G#3	0.45	0.27	1.34	0.33	0.62	0.13	0.06	2.63	155.31	0.52	10.48	0.52	1.44	190.06	1.28	OOR <	OOR	0.01	5.68	OOR <	OOR <	OOR <	1.59	OOR <
LBO D614G#4	0.45	0.31	2.04	0.33	0.54	0.13	0.07	5.36	365.09	0.78	5.25	0.65	2.60	237.29	1.36	OOR <	OOR	OOR	10.15	OOR <	OOR <	OOR <	1.59	OOR <
LBO D614G#5	0.45	0.26	1.04	0.33	0.54	0.13	0.06	14.67	249.69	0.55	7.90	0.52	2.60	273.01	1.28	OOR <	OOR	OOR	8.19	OOR <	OOR <	OOR <	1.44	OOR <
LBO D614G#6	0.45	0.26	1.91	0.33	0.58	0.13	0.05	13.98	255.39	0.61	7.03	0.52	5.17	249.31	1.28	OOR <	OOR	0.01	8.19	OOR <	OOR <	OOR <	1.29	OOR <
LBO B117 #1	OOR <	0.57	2.97	0.48	5.46	0.20	0.09	3.45	2,635.53	0.90	13.81	1.15	5.66	358.94	1.52	OOR <	OOR	OOR	9.17	OOR <	OOR <	OOR <	1.29	OOR <
LBO B117 #2	OOR <	0.65	2.97	0.43	5.75	0.16	0.09	4.80	4,057.28	2.79	13.81	1.41	5.66	328.86	1.76	OOR <	OOR	OOR	10.15	OOR <	OOR <	OOR <	1.59	OOR <
LBO B117 #3	OOR <	0.44	1.34	0.48	1.77	0.13	0.03	10.01	680.73	1.07	3.40	0.52	1.44	458.38	1.28	OOR <	OOR	OOR	9.87	OOR <	OOR <	OOR <	3.96	OOR <
LBO B117 #4	OOR <	0.33	1.63	0.33	2.20	0.16	0.06	6.98	718.28	0.93	9.63	1.15	3.14	366.22	1.28	OOR <	OOR	OOR	7.91	OOR <	OOR <	OOR <	8.82	OOR <
LBO B117 #5	OOR <	0.85	2.45	0.53	4.23	0.55	0.08	12.58	3,074.71	1.49	18.67	1.41	4.18	843.77	3.05	OOR <	OOR	OOR	12.42	OOR <	OOR <	OOR <	0.86	OOR <
LBO B117 #6	OOR <	0.49	1.63	0.33	1.54	0.13	0.03	14.12	2,753.06	0.72	5.25	0.52	OOR <	518.14	1.40	OOR <	OOR	OOR	12.14	OOR <	OOR <	OOR <	0.43	OOR <
LBO P.1 #1	OOR <	0.95	3.36	0.60	3.86	0.16	0.13	36.52	6,960.73	1.55	16.26	1.66	6.61	898.21	4.62	OOR <	OOR	OOR	33.69	OOR <	OOR <	OOR <	0.43	OOR <
LBO P.1 #2	0.57	0.53	1.63	0.38	1.04	0.21	0.09	22.31	2,109.41	0.81	12.15	0.90	6.61	753.85	1.84	OOR <	OOR	OOR	16.95	OOR <	OOR <	OOR <	1.29	OOR <
LBO P.1 #3	0.45	0.70	3.99	0.38	3.51	0.16	0.11	23.13	5,302.71	2.48	12.15	1.66	0.81	1,228.28	2.66	OOR <	OOR	OOR	18.45	OOR <	OOR <	OOR <	1.15	OOR <
LBO P.1 #4	OOR <	0.43	1.77	0.33	1.17	0.15	0.08	9.93	1,276.63	1.37	10.48	0.90	5.66	297.79	1.28	OOR <	OOR	OOR	11.85	OOR <	OOR <	OOR <	2.17	OOR <
LBO P.1 #5	OOR <	0.47	1.48	0.33	1.71	0.13	0.04	7.60	2,898.18	0.84	10.48	OOR <	4.68	298.03	1.28	OOR <	OOR	OOR	13.57	OOR <	OOR <	OOR <	1.00	OOR <
LBO P.1 #6	5.82	0.82	2.97	0.33	3.05	0.16	0.12	26.09	5,689.49	2.64	13.81	2.03	8.45	598.51	3.44	OOR <	OOR	OOR	23.85	OOR <	OOR <	OOR <	1.29	OOR <
LBO Delta #1	0.85	1.14	3.23	1.40	2.77	0.35	0.10	22.06	3,747.80	2.82	17.06	0.90	7.08	876.39	4.69	OOR <	OOR	0.05	57.97	OOR <	OOR <	OOR <	1.29	OOR <
LBO Delta #2	OOR <	0.95	2.97	0.43	1.93	0.54	0.09	43.65	8,023.50	5.00	21.84	0.65	3.66	990.02	4.62	OOR <	OOR	OOR	38.49	OOR <	OOR <	OOR <	1.15	OOR <
LBO Delta #3	OOR <	0.67	2.32	0.53	3.79	0.13	0.08	15.83	4,810.53	2.64	5.25	0.65	9.78	433.08	3.01	OOR <	OOR	OOR	26.70	OOR <	OOR <	OOR <	2.77	OOR <
LBO Delta #4	0.57	0.95	4.35	0.90	1.99	0.79	0.07	64.29	6,919.44	8.03	30.33	2.40	8.45	1,412.45	6.34	OOR <	OOR	OOR	57.14	OOR <	OOR <	OOR <	0.72	OOR <
LBO Delta #5	OOR <	1.12	4.23	0.77	3.74	0.18	0.11	33.61	6,595.41	8.34	13.81	3.13	8.45	883.71	3.55	OOR <	OOR	OOR	24.16	OOR <	OOR <	OOR <	0.72	OOR <
LBO Delta #6	OOR <	0.88	3.36	0.48	2.79	0.41	0.08	33.46	4,831.64	8.91	15.45	0.90	5.66	508.34	3.63	OOR <	OOR	0.01	22.59	OOR <	OOR <	OOR <	0.72	OOR <
LBO BA.1 #1	2.64	0.88	2.71	0.38	6.29	1.23	0.07	46.03	8,142.75	5.34	18.67	1.66	6.61	1,917.95	12.54	OOR <	OOR	0.01	70.13	OOR <	OOR <	OOR <	1.44	OOR <
LBO BA.1 #2	OOR <	0.92	2.97	0.55	3.05	0.97	0.07	104.33	6,767.03	15.41	12.98	1.90	7.54	1,247.53	5.59	OOR <	OOR	OOR	29.60	OOR <	OOR <	OOR <	1.59	OOR <
LBO BA.1 #3	OOR <	1.01	2.85	0.53	3.38	0.69	0.06	25.27	5,666.72	5.59	17.06	1.03	6.61	696.86	4.46	OOR <	OOR	OOR	33.19	OOR <	OOR <	OOR <	0.15	OOR <
LBO BA.1 #4	OOR <	0.93	3.86	0.38	3.43	1.29	0.13	81.01	8,068.49	5.31	15.45	2.40	8.90	1,425.21	5.41	OOR <	OOR	OOR	36.50	OOR <	OOR <	OOR <	0.43	OOR <
LBO BA.1 #5	OOR <	0.82	2.97	0.43	1.74	0.34	0.08	14.20	5,162.28	4.57	13.81	2.15	3.66	1,078.60	4.99	OOR <	OOR	OOR	49.14	OOR <	OOR <	OOR <	1.00	OOR <
LBO BA.1 #6	OOR <	0.93	3.23	0.48	1.57	1.43	0.08	37.14	7,044.47	3.80	12.15	2.15	8.45	610.17	5.97	OOR <	OOR	OOR	60.80	OOR <	OOR <	OOR <	1.00	0.09
LBO BA.2 #1	OOR <	0.85	2.18	0.33	1.17	0.48	0.11	21.24	5,609.36	0.96	17.87	0.90	0.03	652.51	3.82	OOR <	OOR	OOR	41.82	OOR <	OOR <	OOR <	0.43	OOR <
LBO BA.2 #2	0.04	1.11	3.99	0.82	4.83	1.15	0.18	30.38	8,528.33	6.93	23.40	3.13	10.22	2,095.98	8.81	OOR <	OOR	OOR	53.14	OOR <	OOR <	OOR <	2.17	OOR <
LBO BA.2 #3	OOR <	0.39	1.63	0.33	0.91	0.13	0.06	2.16	658.24	2.15	4.33	0.65	OOR <	351.66	1.28	OOR <	OOR	OOR	5.82	OOR <	OOR <	OOR <	0.86	OOR <
LBO BA.2 #4	OOR <	0.30	1.63	0.33	0.70	0.21	0.05	5.75	572.07	1.73	1.44	OOR <	3.14	391.52	1.28	OOR <	OOR	OOR	4.85	OOR <	OOR <	OOR <	0.72	OOR <
LBO BA.2 #5	OOR <	0.30	1.34	0.33	0.21	0.13	0.08	8.69	527.09	1.79	5.25	OOR <	0.81	533.30	1.28	OOR <	OOR	OOR	5.68	OOR <	OOR <	OOR <	0.15	OOR <

The ARRIVE Essential 10: Compliance Questionnaire

Use this questionnaire to evaluate how well a manuscript complies with the ARRIVE Essential 10. It can be applied to any manuscript describing comparative experiments in living animals, by assessors such as journal staff, editors, or peer reviewers.

Item	Question(s)	Answers
1 Study Design	Are all experimental and control groups clearly identified?	☒ Yes, for at least one experiment<br>☐ No
	Is the experimental unit (e.g. an animal, litter or cage of animals) clearly identified?	☒ Yes, for at least one experiment<br>☐ No
2 Sample Size	Is the exact number of experimental units in each group at the start of the study provided (e.g. in the format '*n*=')?	☒ Yes, for at least one experiment<br>☐ No
	Is the method by which the sample size was chosen explained?	☐ Yes, for at least one experiment<br>☒ No
3 Inclusion & Exclusion Criteria	Are the criteria used for including and excluding animals, experimental units, or data points provided?	☒ Yes, for at least one experiment<br>☐ No
	Are any exclusions of animals, experimental units, or data points reported, or is there a statement indicating that there were no exclusions?	☒ Yes, for at least one analysis<br>☐ No
4 Randomisation	Is the method by which experimental units were allocated to control and treatment groups described?	☒ Yes, for at least one experiment<br>☐ No
5 Blinding	Is it clear whether researchers were aware of, or blinded to, the group allocation at any stage of the experiment or data analysis?	☒ Yes, for at least one experiment<br>☐ No
6 Outcome Measures	For all experimental outcomes presented, are details provided of exactly what parameter was measured?	☒ Yes, for at least one experiment<br>☐ No
7 Statistical Methods	Is the statistical approach used to analyse each outcome detailed?	☒ Yes, for at least one analysis<br>☐ No
	Is there a description of any methods used to assess whether data met statistical assumptions?	☐ Yes, for at least one analysis<br>☐ No<br>☐ Not applicable
8 Experimental Animals	Are all species of animal used specified?	☒ Yes, for at least one experiment<br>☐ No
	Is the sex of the animals specified?	☒ Yes, for at least one experiment<br>☐ No<br>☐ Not applicable to species
	Is at least one of age, weight or developmental stage of the animals specified?	☒ Yes, for at least one experiment<br>☐ No
9 Experimental Procedures	Are both the timing and frequency with which procedures took place specified?	☒ Yes, for at least one experiment<br>☐ No
	Are details of acclimatisation periods to experimental locations provided?	☒ Yes, for at least one experiment<br>☐ No
10 Results	Are descriptive statistics for each experimental group provided, with a measure of variability (e.g. mean and SD, or median and range)?	☒ Yes, for at least one experiment<br>☐ No<br>☐ Not applicable to the type of data collected
	Is the effect size and confidence interval provided?	☐ Yes, for at least one experiment<br>☐ No<br>☒ Not applicable to the type of analysis used

ARRIVE

NC3Rs National Centre for the Replacement Refinement & Reduction of Animals in Research

Notes on questionnaire design

The ARRIVE guidelines are a useful resource for authors preparing manuscripts describing animal research, and also provide a framework to evaluate the transparency of those manuscripts. To assess reporting quality, numerous studies have in the past sought to operationalise reporting guidelines (including ARRIVE). Typically, this involves scoring a manuscript's degree of compliance with guideline items in a binary fashion (e.g. an item is either not reported or reported) [1-3], a graded fashion (e.g. not, partially, or completely reported) [4,5], or a combination of the two [6].

This questionnaire has been designed to be as concise and user-friendly as possible. The number of questions used to assess a manuscript's compliance has been kept to a minimum, and in most cases each question is designed to be answered in a binary fashion. Compliance with some Essential 10 sub-items is inherently impossible to judge in this way, instead requiring a subjective judgement on the level of detail provided. For this reason, not all sub-items are represented by a question in this questionnaire.

To facilitate binary answers, it has been necessary to identify the minimum information in a manuscript sufficient to comply with each question. The strengths of this approach include the relatively short length of the questionnaire (and the correspondingly low time burden of using it), and the avoidance of ambiguity that would arise from a graded answering system, in which an intermediate score (e.g. 'partially/insufficiently reported') could denote a number of distinct deficiencies in compliance with an item (e.g. either only part of the item was complied with, or only the reporting of some experiments in the manuscript complied with the item.)

Limitations of this approach centre on the necessity to identify the minimum information sufficient to comply with each question. In some cases, this has resulted in questions that require a guideline sub-item's criteria to have been fulfilled in the reporting of only one experiment in a manuscript. As a result, not all experiments in a manuscript may be described in a way that fulfils that criterion, despite the manuscript being considered to comply with the guidelines overall.

References

1. Hair *et al* (2020). *Res Integ Peer Rev*. doi: 10.1186/s41073-019-0069-3
2. Tihanyi *et al* (2019). *J Surg Res*. doi: 10.1016/j.jss.2018.10.038
3. Zhao *et al* (2020). *BMC Vet Res*. doi: 10.1186/s12917-020-02664-1
4. Han *et al* (2017). *Plos One*. doi: 10.1371/journal.pone.0183591
5. Chatzimanouil *et al* (2019). *J Am Soc Nephrol*. doi: 10.1681/ASN.2018050515
6. Leung *et al* (2018). *Plos One*. doi: 10.1371/journal.pone.0197882
